# A microscopic simulation model for pedestrian-pedestrian and pedestrian-vehicle interactions at crosswalks

**DOI:** 10.1371/journal.pone.0180992

**Published:** 2017-07-17

**Authors:** Manxia Liu, Weiliang Zeng, Peng Chen, Xuyi Wu

**Affiliations:** 1 Shenzhen Institute of Standards and Technology, Futian District, Shenzhen, P.R., China; 2 Institution of Materials and Systems for Sustainability, Nagoya University, Furo-cho, Chikusa, Nagoya, Japan; 3 Department of Transportation Science and Engineering, Beihang University, Haidian District, Beijing, P.R. China; Rijksuniversiteit Groningen, NETHERLANDS

## Abstract

This study aims to develop a microscopic pedestrian behavior model considering various interactions on pedestrian dynamics at crosswalks. Particularly, we take into account the evasion behavior with counter-flow pedestrians, the following behavior with leader pedestrians, and the collision avoidance behavior with vehicles. Aerial video data at one intersection in Beijing, China are extracted for model calibration. A microscopic calibration approach based on maximum likelihood estimation is applied to estimate the parameters of a modified social force model. Finally, we validate step-wise speed, step-wise acceleration, step-wise direction change, crossing time and lane formation phenomenon by comparing the real data and simulation outputs.

## Introduction

Studying the self-organization phenomena of pedestrian crowd is an active subject in transportation science. To date, pedestrian behavior modeling has attracted considerable attentions [1–7]. A better understanding of the interaction behavior would help to improve microscopic simulation and thus allow more accurate prediction of their behavior for various situations. This also helps to evaluate the service and safety level on pedestrian related traffic, such as pedestrian movement in urban streets and crosswalks.

Generally, existing pedestrian behavior models can be classified into three categories: macroscopic, mesoscopic and microscopic models. In the last decades, mesoscopic and microscopic models have attracted much attention because they enable to offer a more detailed analysis on pedestrian behavior.

The mesoscopic models are usually based on kinetic theory and game theory, in which the microscopic interaction can be represented through a statistical distribution of the microscopic position and velocity. One of the motivations of applying kinetic theory is to model the complexity issues of living systems such as crowds. Following the kinetic theory, Bellomo et al developed a simulation model representing the dynamics of collective behaviors [5, 8]. Degond et al introduced a hierarchy of kinetic and macroscopic models derived from a heuristic description at the micro-scale, and they developed an analogy between a Local Thermodynamically Equilibrium and Nash equilibria in a game theoretic framework [9]. Wang et al [10] developed two efficient numerical methods for a multiscale kinetic equation in the context of crowd dynamics with emotional contagion, and they particular focused on when the particle characteristics can cross and whose long time behavior is not flocking.

The family of microscopic models includes Cellular Automata [11, 12], social force model [13–15], velocity-based model [16, 17], discrete choice model [18] and lattice gas model [19, 20]. To our knowledge, Cellular Automata model and social force model are popular models for pedestrian dynamics because they are able to describe most of the self-organization phenomena of large crowd. Cellular Automata are microscopic models with grid-based motion decisions, in which a set of rules define the state/occupation of a cell in dependence of the neighborhood of the cell, and a transition matrix is used to update the cell states in successive time steps. However, pedestrians behave flexibly and the choice of next step is unrestricted dynamic, which cannot be fully taken into account by only choosing one option from a limited set of cells. The fluid crowd modeling method of Henderson [21] has been the starting point of the social force model. Later, a magnetic-force model [22] was developed by borrowing a motion equation used for magnetic fields. Based on these concepts, a more robust physical force based model, i.e., social force model [11], was developed and applied to evacuation analysis. The physical force based model makes it possible not only to accurately describe dynamic pedestrian movement in space, but also to reproduce the self-organization phenomenon such as lane formation, stop-and-go waves and turbulence [4].

Pedestrian microscopic simulation has gathered a lot of interest in the modeling of safety analysis of pedestrian infrastructures and crowd evacuation. Lian et al. analyzed collective movement characteristics on a four-directional intersecting flow and they found that putting an obstacle in the center of cross area will improve traffic stability [23]. Duives et al. found that the distance headway, the time headway, the sight angle, the interaction angle, the absolute speed and the number of pedestrians located nearby significantly influence on the strength of the reaction of pedestrians walking within a crowd [24]. Bellomo et al. [25] proposed to view human crowds as a large living system in evacuation dynamics. They found that the dynamics can be subject to the heterogeneous behaviors and social interactions. Ronchi et al [26] developed a multi-agent continuous model for large-scale evacuation safety simulation at music festivals. They found that the evacuation time curves coupled with the visual analysis allowed for identifying the predominant factor affecting evacuation such as delay time and flows through exits. In recent decades, a large number of studies have been focused on bi-directional flow in pedestrian dynamics [27, 28]. The basic characteristics related to fundamental diagrams [27] and self-organization such as lane formation [29, 30] and jamming transition [31], were investigated in experimental ground fields or actual scenes. Interestingly, there is no conclusion yet whether the traffic flow performance is different or not between uni-directional and bi-directional flows. Teknomo found that the two-way traffic performance reduces significantly as the number of pedestrians increase [32]. Lam et al. stated that there is no significant difference between bidirectional flow and unidirectional flow if the densities of the opposite streams are similar [33]. Helbing et al. stated that counter-flows could be more efficient than unidirectional flows [29]. However, they compare average flows without considering the influence of the density. Kretz et al. found that the speed and fluxes did not reduce a lot within bi-directional flow due to the self-organization phenomenon [28].

Crossing behaviors at unsigalized and signalized intersections are critical factors that may result in safety problems. A case study in China showed that the rates of compliance with traffic rules at signalized intersections are influenced by crossing distance, signal timing, and pedestrian volume [34]. Yang et al. [35] developed a microscopic simulation model for pedestrians’ signal non-compliance decision in the mixed traffic in China. Gorrini et al. [36] modeled the elderly inhabitants and risky pedestrian-vehicle interaction on unsignalized intersections. Feng et al. [37] built a microscopic model to simulate crossing behavior in a street to evaluate the service level. Li et al. [38] modified the social force model by considering the required space and the critical gaps with turning vehicles, which makes it possible to describe the stop/go decision to the conflicting vehicles. Anvari et al. [39] simulated the interaction between pedestrian and vehicle by using a rule-based social force model for shared space environments.

In addition, many research focused on the model calibration and validation. Isenhour et al. developed a pedestrian simulation tool for fire evacuation analysis and they recommended seventeen verification tests according to the United States’ National Institute of Standards and Technology [40]. Daamen and Hoogendoorn designed laboratory experiments to estimate parameters of the Nomad pedestrian simulation model for bottlenecks in the evacuation of a building and the simulation results showed that complex walker models can indeed be calibrated by empirical data [41, 42]. Campanella et al. further quantified the validation of the Nomad pedestrian simulation model by combining multi-objective assessments such as average travel times, the speed-density fundamental relation and bottleneck capacity [43]. Seer et al. estimated the model parameters and their distributions with nonlinear regression based on observed trajectories [44].

To develop and calibrate microscopic pedestrian models usually requires accurate and comprehensive trajectory data on individual pedestrian movement. The traditional data acquisition method is to shoot video and apply tools to detect and track the individual movement of each pedestrian. Both manual and automated tracking approaches can be found. Multiple cameras were usually used to shot videos at intersections and manually extracted trajectories of individual pedestrians and turning vehicles [6, 7]. The synchronization among multiple cameras from different views was complicated and required extra effort. To facilitate trajectory extraction process from video, some researches [44–47] conducted control experiments by equipping participants with distinctive wear, e.g., colored hats, for better identification. With the recent price drop of off-the-shelf unmanned aerial vehicle (UAV) products, increasing researchers are exploring the potentials of using UAVs for pedestrian and vehicle detection and tracking. On the basis of the authors’ ongoing work, this study will use UAVs to obtain accurate tracking data from a top-down view for pedestrian behavior model calibration.

In summary, there is growing interest in developing a simulation model for pedestrian dynamic behavior in various scenarios. However, only limited studies shed light on crossing behavior such as interaction with counter-flow pedestrians, turning vehicles, and traffic regulations. For practical applications such as traffic safety assessment at crosswalks, the current pedestrian models for crowd simulation are inadequate. This study aims to fill this gap by developing a microscopic model considering various interaction behaviors among road users at crosswalk. Furthermore, an estimation approach for calibrating the microscopic model based on real trajectory data is proposed. Last, the validation is conducted to confirm the model performance on step-wise location, fundamental diagram and lane formation phenomenon.

## Model description

**Fig 1:**
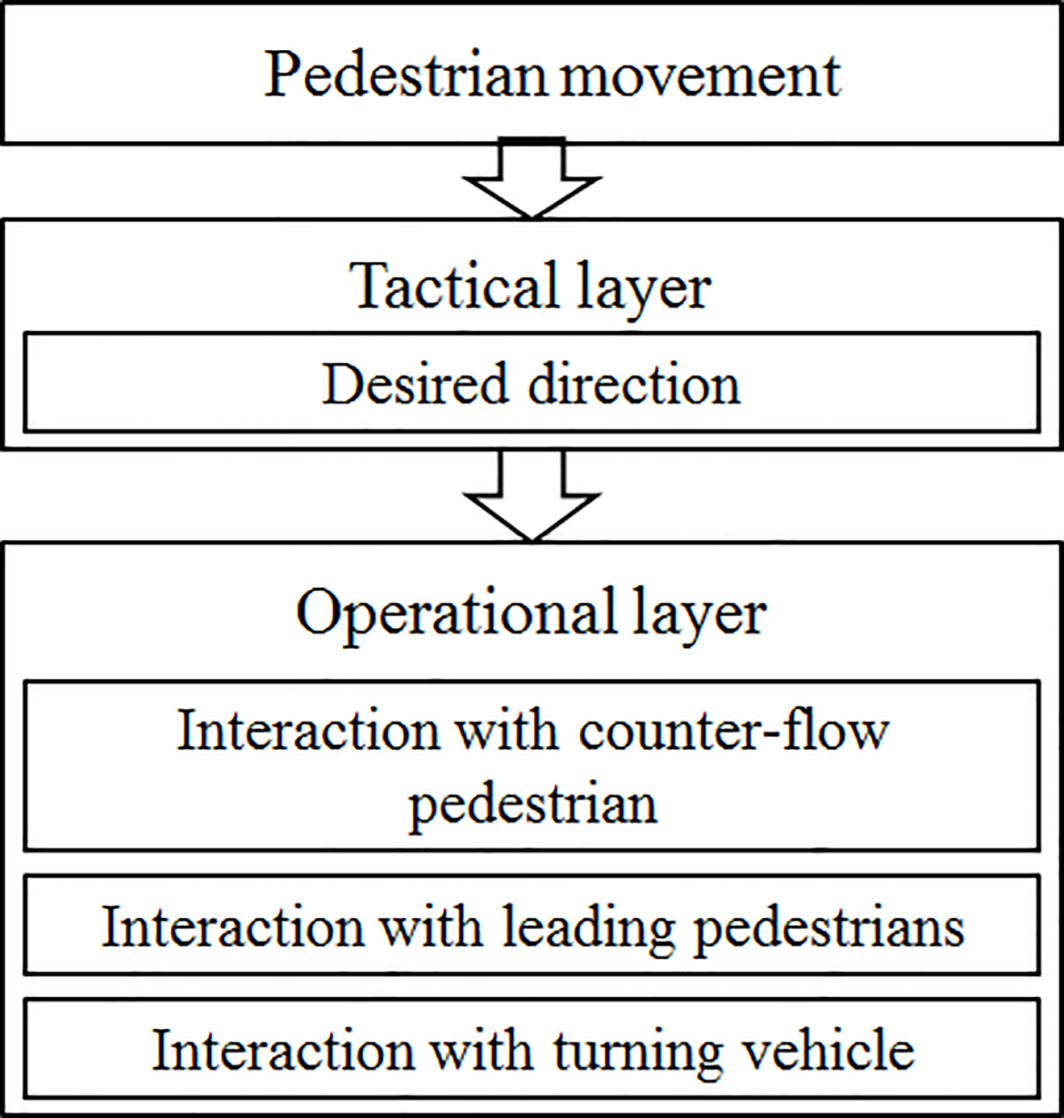
Model framework. The microscopic model includes two layers, i.e., a tactical layer and an operational layer. The desired direction of movement is determined in the tactical layer. The operational layer determines the microscopic behavior when pedestrians interact with other agents.

As shown in Fig 1, the microscopic model includes two layers, i.e., a tactical layer and an operational layer, inspired by Hoogendoorn and Bovy’s conceptual modeling framework [48]. The desired direction of movement is determined in the tactical layer. The operational layer determines the microscopic behavior when pedestrians interact with other agents. In this layer, we assume that the subject pedestrian interacts with other pedestrians and vehicles. The interaction with surrounding pedestrians can be further divided into two types, i.e., interaction with counter-flow pedestrians and interaction with leading pedestrians. A repulsive force is used to represent the interaction with counter-flow pedestrians, while an attractive force is used to represent the interaction with leading pedestrians. The pedestrian-vehicle conflict mechanism is also included in this model. Risk-taking pedestrians might enter the crosswalk even though the vehicle is approaching. We model the “waiting/crossing” behavior with a bi-logit model and develop detour route plan and repulsive force model for pedestrian-vehicle interaction. The detail of the pedestrian behavior in the tactical and the operational layers is introduced in the following sections.

### Desired direction

**Fig 2:**
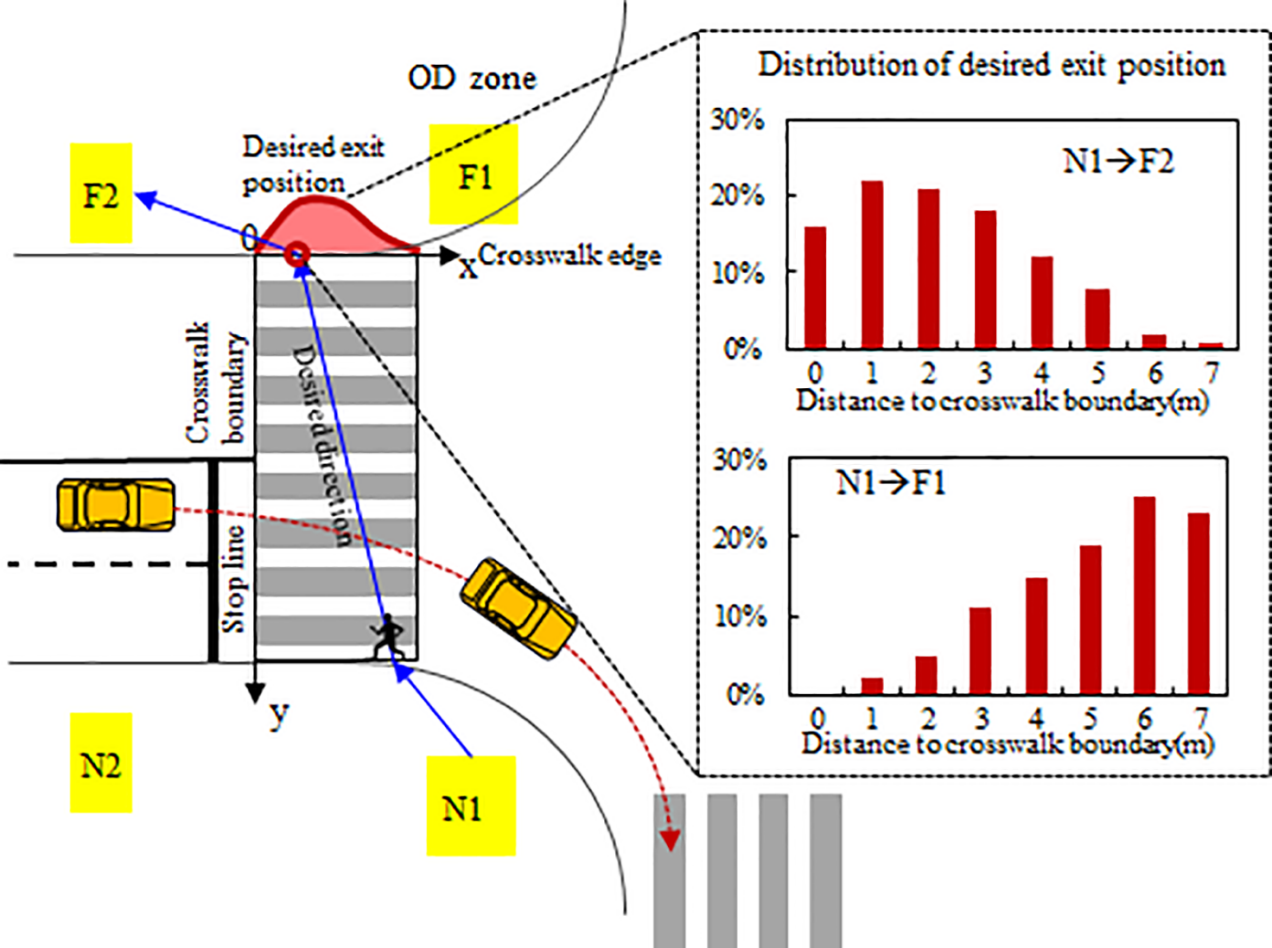
Desired exit position and desired direction. The desired direction is assumed to be determined by the desired exit position of the crosswalk. The desired exit position is defined as the intersecting point of the curve of crosswalk edge and the desired walking trajectory.

**Fig 3:**
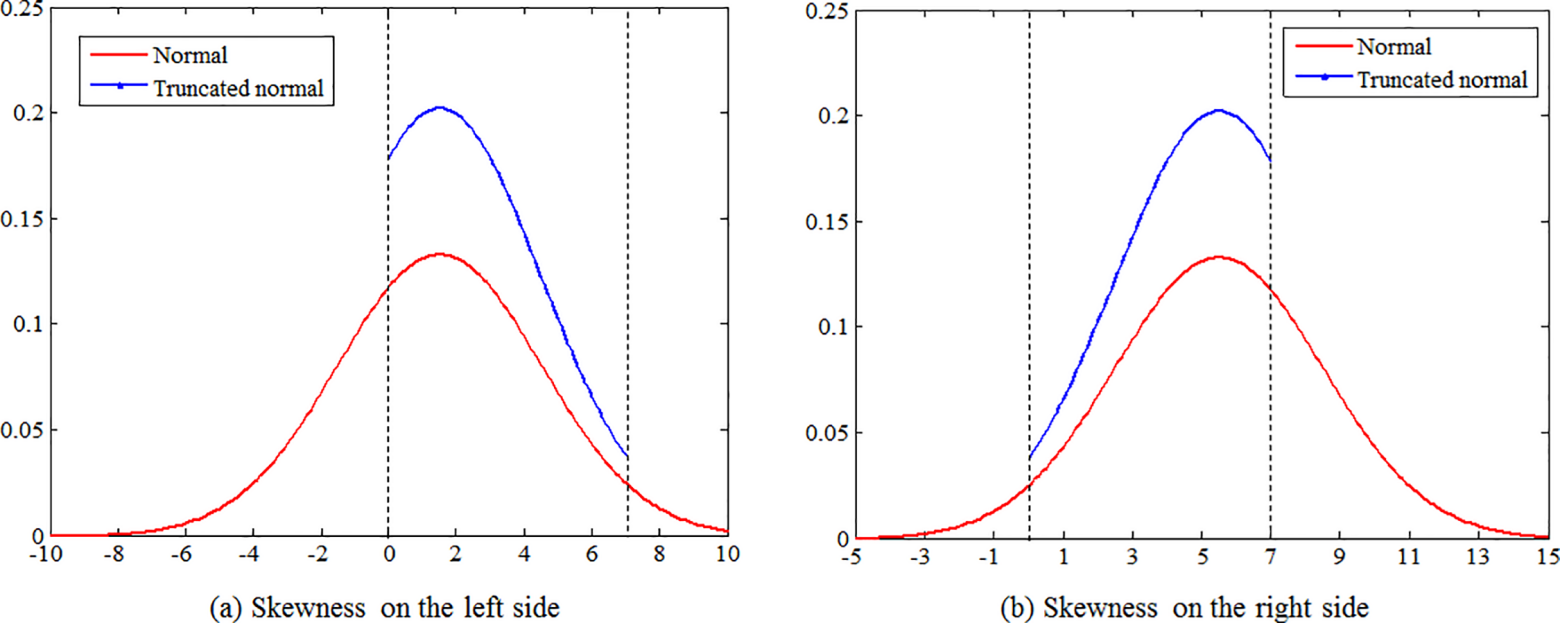
Truncated normal distribution. The concept of truncated normal distribution is able to represent a distribution with arbitrary skewness and a specified range.

As shown in Fig 2, the desired direction is assumed to be determined by the desired exit position of the crosswalk. The desired exit position is defined as the intersecting point of the curve of crosswalk edge and the desired walking trajectory. Assumed that the shape of crosswalk is rectangular, the desired exit position can be represented by using the perpendicular distance to the crosswalk boundary (stop line side) when the pedestrian exits the crosswalk. We assumed that the distribution of exit positions is influenced by origin-destination (OD). More specifically, the exit position distribution is assumed to have a peak on the right side or left side and the side of skewness is dependent on the OD direction. However, most probability distributions (including Gamma, lognormal, and Weibull) cannot represent a distribution with right-side skewness and they assume that the random variable spreads over the whole range of the real number axis, but in practice, the exit position makes sense only within the width of the crosswalk. To fill this gap, the concept of truncated normal distribution is introduced, which is able to represent a distribution with arbitrary skewness and a specified range as shown in Fig 3. The probability density function is given by:

$$f(x;\mu ,\sigma ,a,b)=\frac{f(x)}{\int_{a}^{b}f(x)dx}$$ (1)

$$f(x)=\frac{1}{\sqrt{2\pi }\sigma }e^{-\frac{{\left(x-\mu \right)}^{2}}{2\sigma^{2}}}$$ (2)

$$\int_{a}^{b}f(x)dx=\Phi \left(\frac{b-\mu }{\sigma }\right)-\Phi \left(\frac{a-\mu }{\sigma }\right)$$ (3)

where *x* denotes the exit position; *a* and *b* denote the lower bound and upper bound of the truncated range, respectively, which are constrained by the crosswalk width; *Φ* denotes the cumulative distribution function of a standard normal distribution; *μ* and *σ* are the parameters to be estimated, which denote the mean and standard deviation of the non-truncated distribution, respectively.

We assumed that the distribution of exit position is influenced by the OD direction, crosswalk length *d*_*l*_, crosswalk width *d*_*w*_ and pedestrian density *ρ*. *μ* and *σ* can be expressed as the following regression functions.

$$\mu =b_{0}M_{0}+b_{1}M_{1}+b_{2}M_{2}+b_{3}d_{l}+b_{4}d_{w}+b_{5}\rho +b_{6}$$ (4)

$$\sigma =c_{0}M_{0}+c_{1}M_{1}+c_{2}M_{2}+c_{3}d_{l}+c_{4}d_{w}+c_{5}\rho +c_{6}$$ (5)

where *M*_0_, *M*_1_, and *M*_2_ are the dummy variables representing eight directions of the OD on a crosswalk; *b*_0_,…,*b*_6_,*c*_0_,…,*c*_6_ are the model coefficients to be estimated.

### Driving force

The step-wise decision process of movement is assumed to include two steps. First, the pedestrian selects the velocity direction based on the desired direction and subsequently the desired speed. Second, the pedestrian adjusts the speed to avoid the conflict with other pedestrians and vehicles.

Pedestrians are assumed to move with individual desired speed $v_{\alpha }^{d}$ and desired direction ${\vec{e}}_{\alpha }$ to the next destination. The desired direction ${\vec{e}}_{\alpha }$ is determined by the current position ${\vec{P}}_{\alpha }$ and the exit position ${\vec{P}}_{e}$ at crosswalk. A deviation of the current speed vector ${\vec{v}}_{\alpha }$ from the desired speed vector $v_{\alpha }^{d}{\vec{e}}_{\alpha }$ leads to a force to recover to the desired speed within a certain relaxation time *τ*_*α*_.

$${\vec{F}}_{d}=\frac{1}{\tau_{\alpha }}(v_{\alpha }^{d}{\vec{e}}_{\alpha }-{\vec{v}}_{\alpha })$$ (6)

$${\vec{e}}_{\alpha }=\frac{{\vec{P}}_{e}-{\vec{P}}_{\alpha }}{\left\|{\vec{P}}_{e}-{\vec{P}}_{\alpha }\right\|}$$ (7)

According to the empirical analysis [29], the desired speed $v_{\alpha }^{d}$ is approximately normal distribution. To compensate for delays at signalized crosswalk, the desired speed is assumed to increase in the course of waiting time due to the traffic light. Assumed that the desired speed is influenced by the waiting time *t*_*w*_ and pedestrian density *ρ*, we formulate the regression function for desired speed as follows.

$$v_{\alpha }^{d}=a_{0}t_{w}+a_{1}\rho +a_{2}+e$$ (8)

$$e∼N(0,{\left(\sigma_{\alpha }^{d}\right)}^{2})$$ (9)

where *a*_0_, *a*_1_, and *a*_2_ are the model coefficients to be estimated; $\sigma_{\alpha }^{d}$ is the standard deviation of the error term.

### Interaction with counter-flow pedestrians

**Fig 4:**
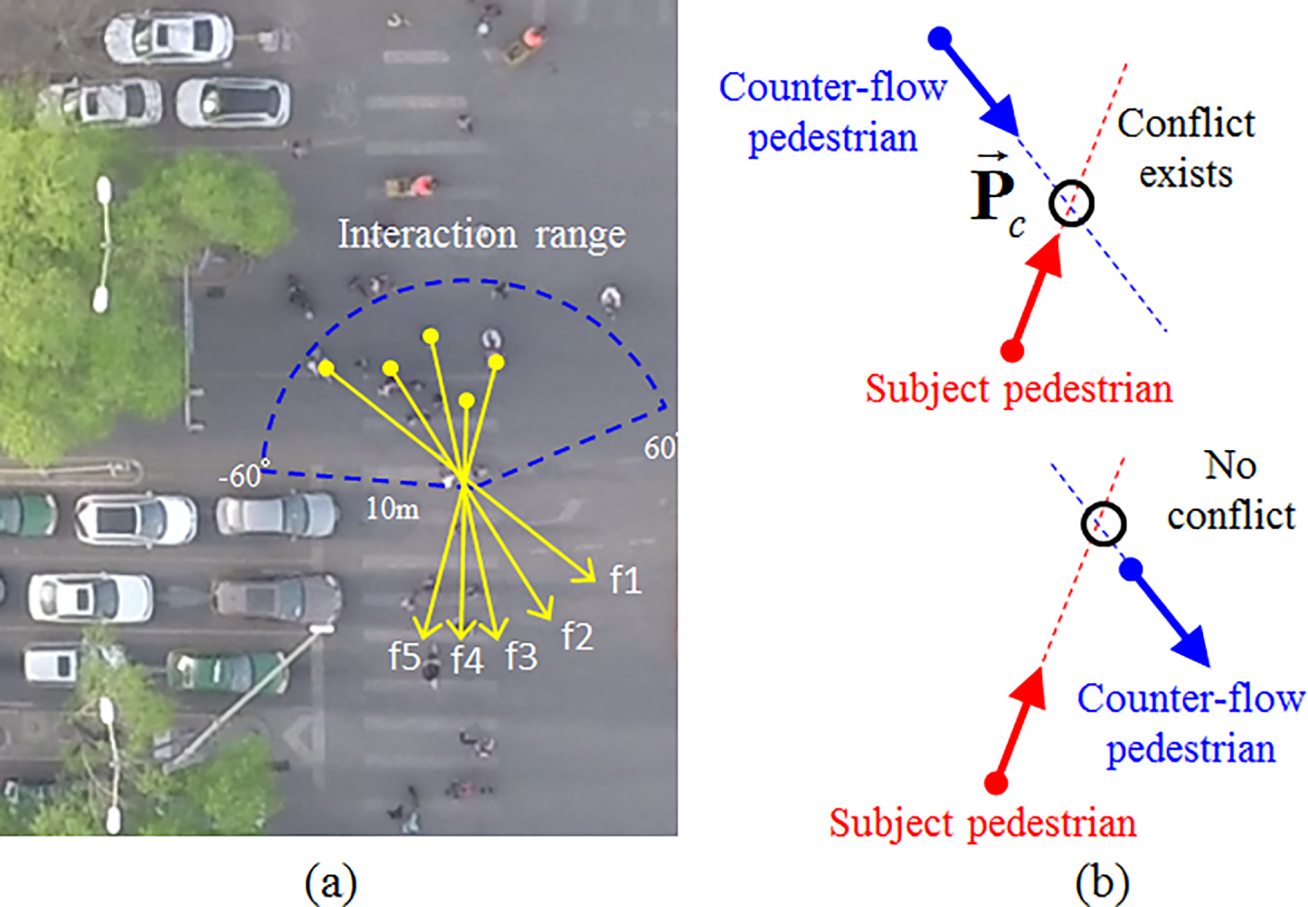
Collision avoidance with counter-flow pedestrians. (a) It is usually assumed that the magnitude of the repulsive force increases monotonically as the relative distance decreases. (b) It shows the case of valid and invalid conflicts.

**Fig 5:**
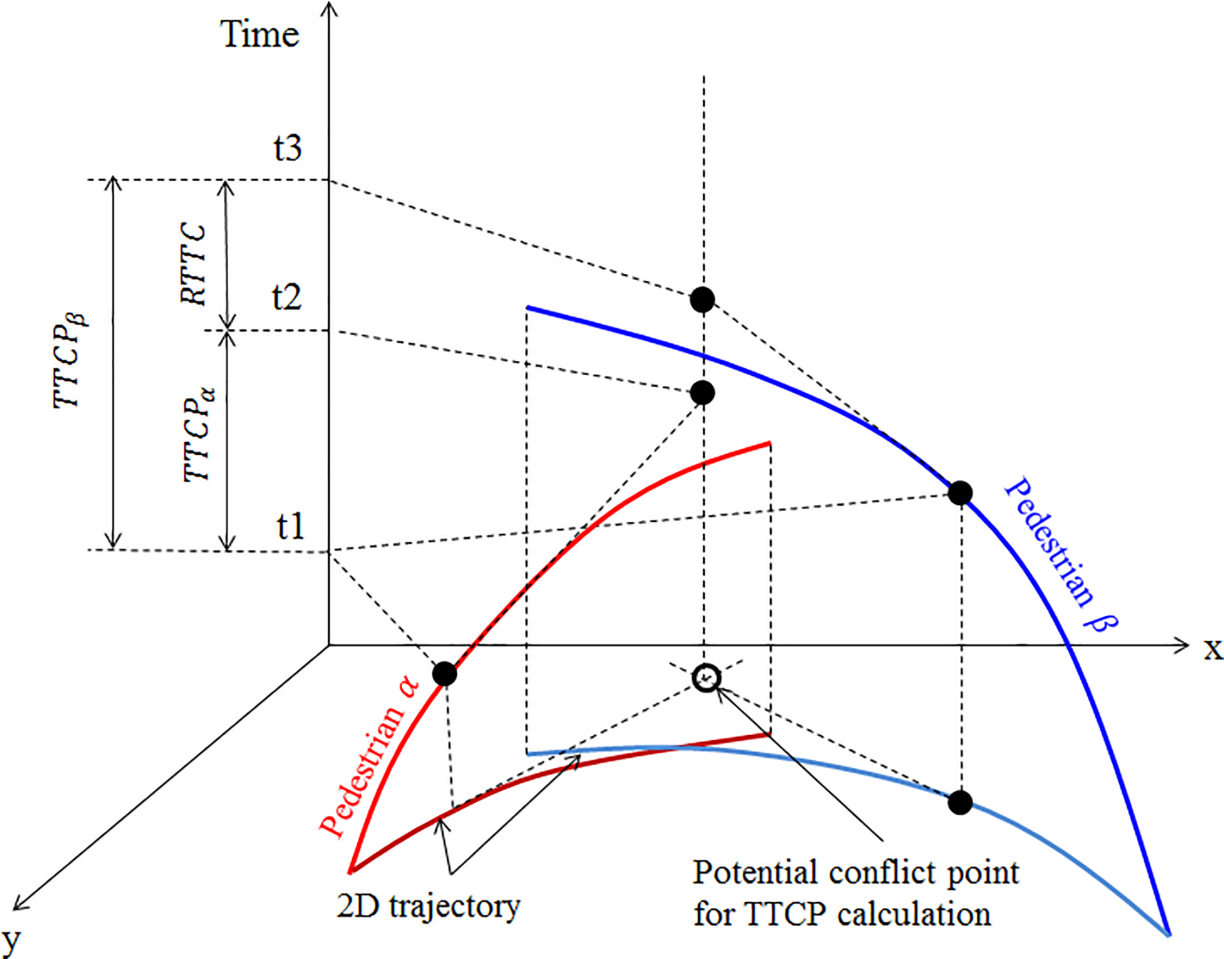
Definition of TTCP. The relative time to collision (RTTC) is defined as the time difference between the first road user arriving at the potential conflicting location and the second road user arriving at this location if they keep their current speeds.

According to the social force theory [13], each conflicting pedestrian within the subject pedestrian’s visual range is assumed to generate a repulsive force to the subject pedestrian, as shown in Fig 4(A). In the original social force model, it is usually assumed that the magnitude of the repulsive force increases monotonically as the relative distance decreases. However, this assumption is not realistic, considering the fact that the repulsive effect might be quite weak if no potential conflict exists or the relative time to potential conflict point will be quite long. Fig 4(B) shows the case of valid and invalid conflicts. The potential conflict does not exist if their future trajectories have no intersect by keeping their current walking directions. The relative time to the potential conflict should be infinite and the repulsive effect does not exist if two pedestrians stop walking even though they are very close. As shown in Fig 5, the relative time to collision (RTTC) is defined as the time difference between the first road user arriving at the potential conflicting location and the second road user arriving at this location if they keep their current speeds. Here, we use the concept of time to conflict point (TTCP) to identify the valid conflict situation. TTCP is defined as the expected time for two pedestrians to pass the intersection point of their trajectories if they keep their current speeds and directions. The TTCP for the subject pedestrian *α* and the conflicting pedestrian *β* can be given as follows.

$${TTCP}_{\alpha }=\frac{\left\|{\vec{P}}_{c}-{\vec{P}}_{\alpha }\right\|}{\left\|{\vec{v}}_{\alpha }\right\|}cos\langle {\vec{v}}_{\alpha },{\vec{P}}_{c}-{\vec{P}}_{\alpha }\rangle$$ (10)

$${TTCP}_{\beta }=\frac{\left\|{\vec{P}}_{c}-{\vec{P}}_{\beta }\right\|}{\left\|{\vec{v}}_{\beta }\right\|}cos\langle {\vec{v}}_{\beta },{\vec{P}}_{c}-{\vec{P}}_{\beta }\rangle$$ (11)

Where

${\vec{P}}_{\beta }$ is the current position of the conflicting pedestrian *β*;
${\vec{P}}_{c}$ is the intersection point of the two pedestrian trajectories.

The positive values of TTCPs of both pedestrians indicate that the conflict exists, while the negative values indicate that one pedestrian had passed the conflict point and no potential collision will occur.

Accordingly, TTCP can be formulated as follows.

$$T_{\alpha \beta }=\left\{ \begin{array}{l} |{TTCP}_{\alpha }-{TTCP}_{\beta }|,\;if\;{TTCP}_{\alpha }>0\;and\;{TTCP}_{\beta }>0\\ +\infty ,\;otherwise \end{array}\right.$$ (12)

The relative time (*T*_*αβ*_) to the conflict point instead of the relative distance is considered as the influential factors to the repulsive force. The repulsive force (${\vec{F}}_{\alpha \beta }^{r}$) of conflicting pedestrians can be presented as follows.

$${\vec{F}}_{\alpha \beta }^{r}=\sum_{i=1}^{n}A_{\beta }^{r}e^{\frac{-T_{\alpha \beta }}{B_{\alpha \beta }^{r}}}{\vec{n}}_{\beta_{i}}$$ (13)

where $A_{\beta }^{r}$ is the interaction strength coefficient, $B_{\alpha \beta }^{r}$ is the interaction range coefficient for the relative time, *n* is the number of conflicting pedestrians, ${\vec{n}}_{\beta_{i}}$ is the normalized vector which is pointing from pedestrian *β*_*i*_ to *α*.

### Interaction with leading pedestrians

We observed that pedestrians preferred to follow the leading pedestrians and joined the group with similar walking directions to avoid intensive interaction with the counter-flow pedestrians. In crowded situations, pedestrians can keep a stable speed and move smoothly by following the leading pedestrians without interactions with the counter-flow pedestrians frequently. Such leader-follower behavior naturally caused the lane formation phenomenon especially when the pedestrian flow became crowded. Even though the lane formation could be also generated without explicitly considering the leader-follower behavior, the generated “lanes” were weak and were easy to breakdown in our original model. To present the collective behavior that result in the swarming effect when the pedestrian density become high at signalized intersections, the attractive force from the leading pedestrians is also formulated.

**Fig 6:**
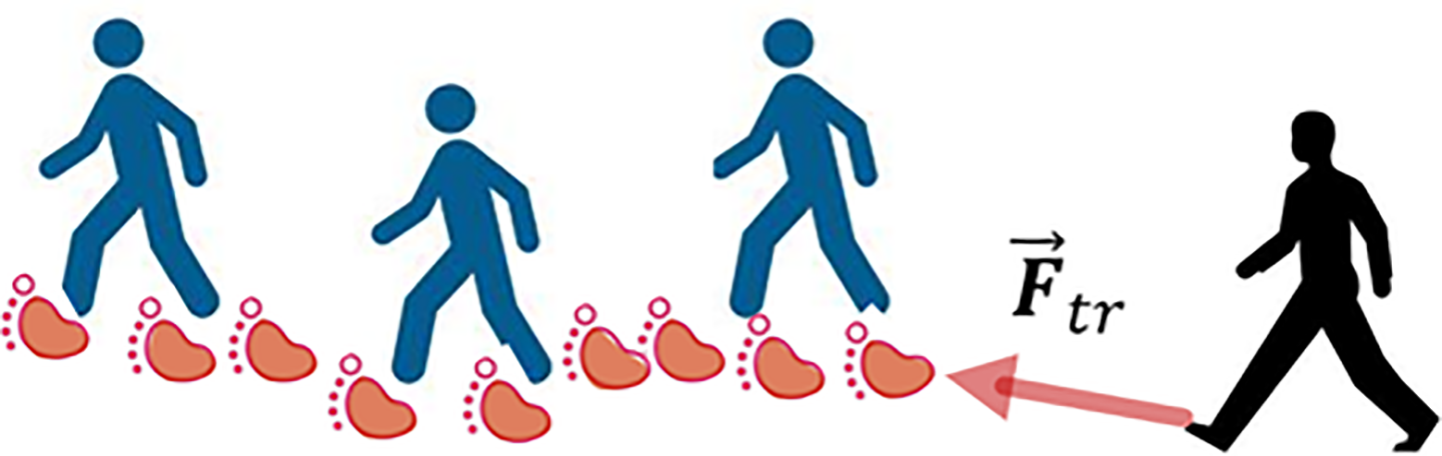
Trail of “footprint”. The subject pedestrian will be attracted by the “footprints” of the pedestrians ahead with the same movement direction.

As shown in Fig 6, it is assumed that the subject pedestrian will be attracted by the “footprints” of the pedestrians ahead with the same movement direction [49]. These “footprints” will disappear in the course of time with a rate 1/*T* where *T* can be regarded as the lifecycle of “footprints”. Different from the repulsive force, the function of the attractive force is not only related to the distance to “footprints”, but also related to the lifecycle of “footprints”. According to Helbing’s study [49], the attractive force (${\vec{F}}_{tr}$) generated from “footprints” can be formulated as follows.

$${\vec{F}}_{tr}=\Delta t\sum_{n=1}^{T/\Delta t}\sum_{\beta }A_{\beta }^{a}exp(-B_{\beta }^{a}\|{\vec{P}}_{\alpha }(t)-{\vec{P}}_{\beta_{i}}(t-n\Delta t)\|-\frac{n\Delta t}{T}){\vec{n}}_{\alpha \beta_{i}}(t-n\Delta t)$$ (14)

where Δ*t* denotes the discretization interval of lifecycle *T*, *n* is the number of interval, ${\vec{P}}_{\beta_{i}}(t-n\Delta t)$ is the position of the “footprint” of pedestrian *β*_*i*_ at time (*t*−*n*Δ*t*), ${\vec{n}}_{\alpha \beta_{i}}(t-n\Delta t)$ is the normalized vector pointing from pedestrian *a* to the “footprint” of pedestrian *β*_*i*_ at time (*t*−*n*Δ*t*), $A_{\beta }^{a}$ and $B_{\beta }^{a}$ are the coefficients to be estimated.

### Interaction with turning vehicles

Pedestrian-vehicle conflicts frequently occur due to the shared signal phase and risk-taking behavior. Drivers are usually required to yield to crossing pedestrians in a right-turn-permitted signal phase (right-hand traffic). However, in developing countries such as China, certain drivers might undertake risky behavior such as entering the crosswalk even if pedestrians are approaching. On the other hand, some risk-taking pedestrians enter the crosswalk before the green light or at the beginning of the red light, which also increase the severe conflict with the vehicles. Since this study mainly focuses on the crossing behavior of pedestrian, the behavior of vehicle is not discussed here. To model the pedestrian behavior during the pedestrian-vehicle interaction process, a three-layer strategy is implemented. In the upper layer, the pedestrian can choose to wait or cross to avoid the conflict when a vehicle is approaching. If the pedestrian chooses to cross, he/she will plan a detour route to avoid the potential collision in the middle strategy layer. Then, a repulsive force from the turning vehicle further acts on the pedestrian moving behavior in the lower layer.

**Fig 7:**
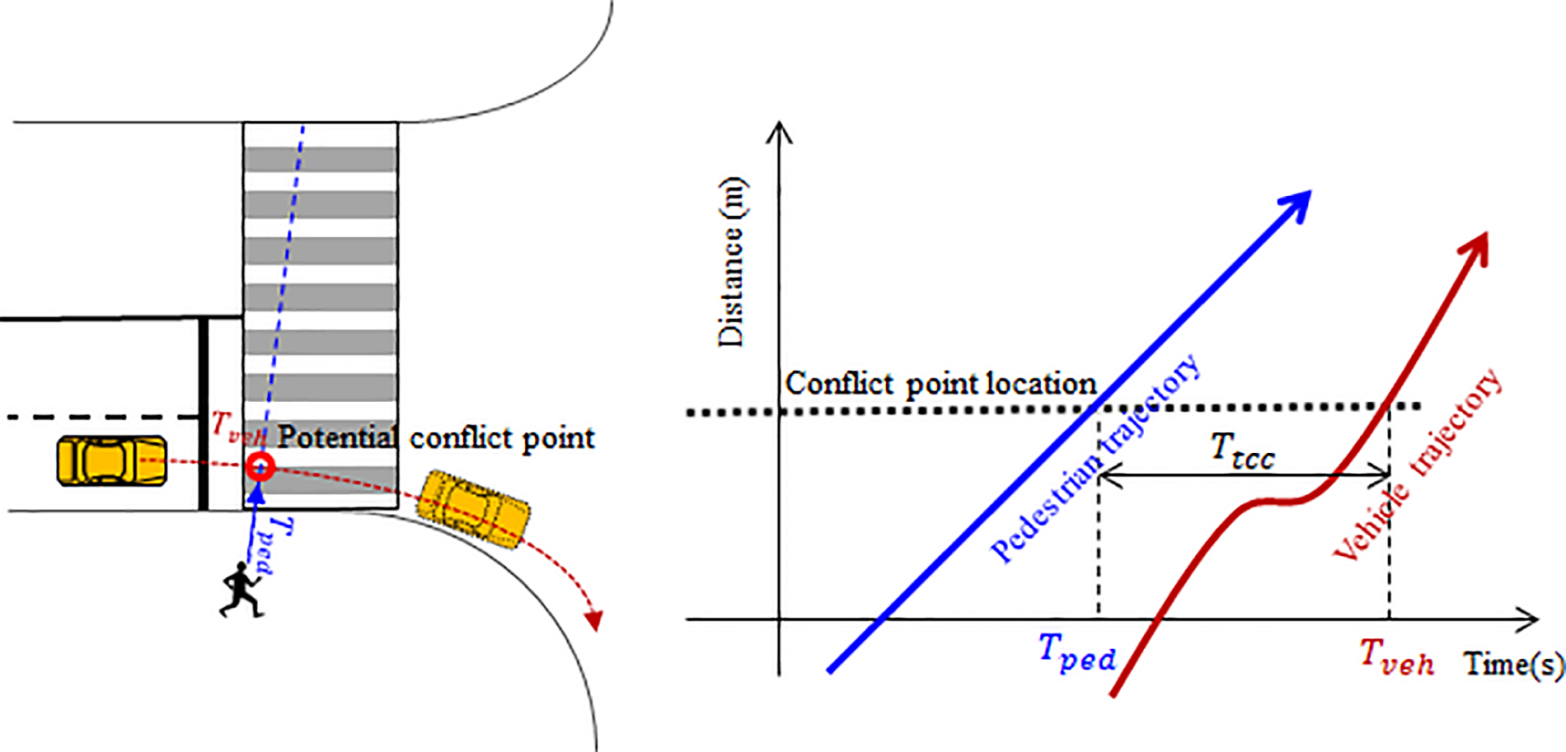
Pedestrian-vehicle conflict. There are two types of pedestrian giving-way maneuvers when the pedestrian-vehicle conflict occurs: waiting until the vehicle passes by and crossing before the vehicle passes by.

In the upper layer of decision making, we assume that there are two types of pedestrian giving-way maneuvers when the pedestrian-vehicle conflict occurs: waiting until the vehicle passes by and crossing before the vehicle passes by. We expect that the probability of choosing crossing is lower if the pedestrian reaches the potential conflict point later than the vehicle. Therefore, a binary logit model can be utilized to describe this behavior, in which the utility function can be formulated by the relative time to the potential conflict point as shown in Fig 7. The “waiting/crossing” strategy is formulated as follows.

$$P_{r}(crossing)=\frac{exp(K)}{1+exp(K)}$$ (15)

$$K=e_{0}T_{ttc}+e_{1}$$ (16)

$$T_{ttc}=T_{veh}-T_{ped}$$ (17)

where *P*_*r*_(*crossing*) is the probability of choosing “crossing”, *T*_*ttc*_ is the relative time to the potential conflict point, *T*_*veh*_ is the time to the potential conflict point for the vehicle, *T*_*ped*_ is the time to the potential conflict point for the pedestrian, *e*_0_ and *e*_1_ are the coefficients to be estimated.

**Fig 8:**
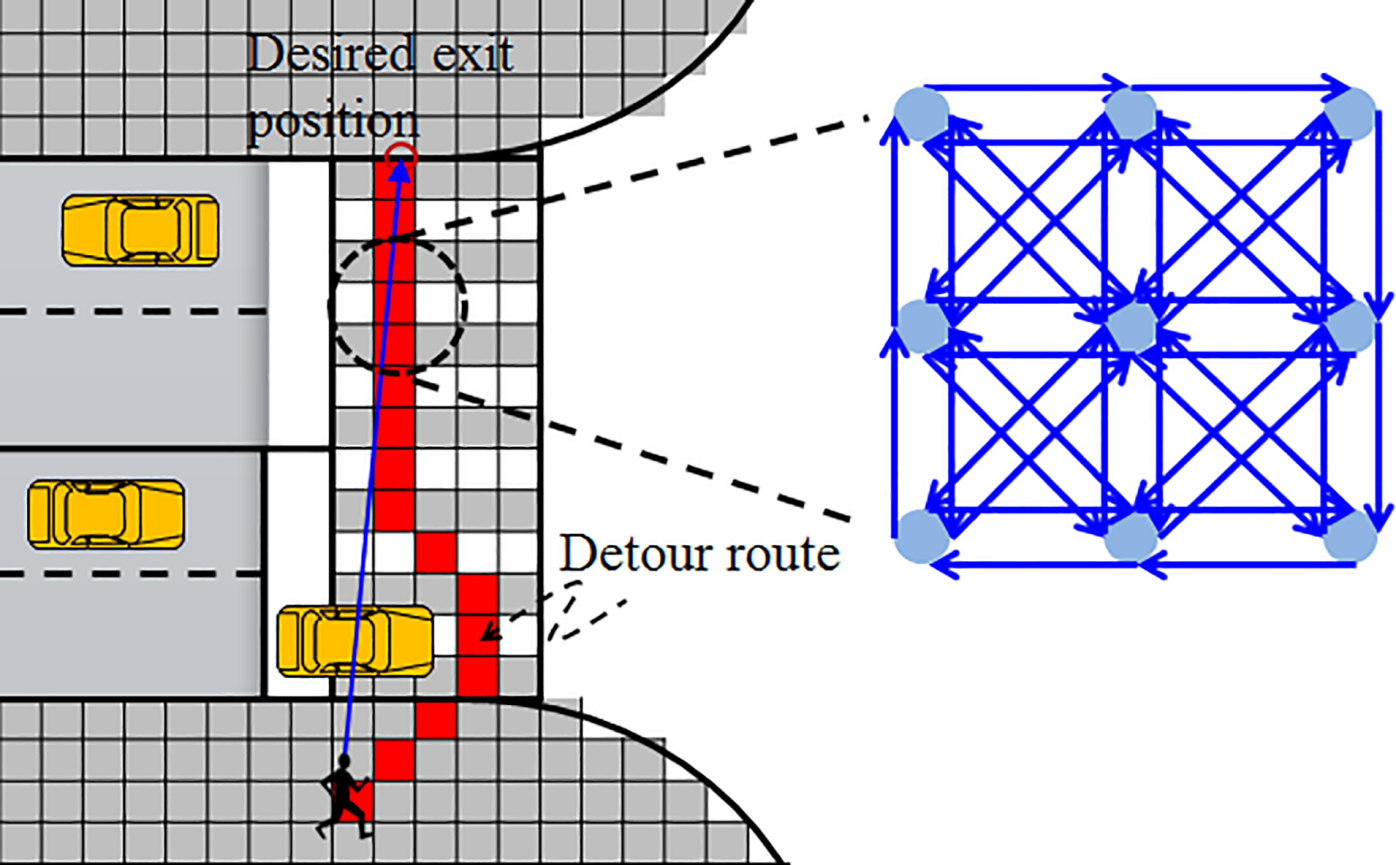
Middle layer: Route plan. The walking space is divided into separate cells where each cell is connected to each other by directed links.

In the middle strategy layer, we assume that pedestrians can identify possible routes to avoid the collision with vehicles based on the occupied location of conflicting vehicles. Pedestrians move through the desired route from their origin to destination via intermediate destinations. A link-node network as shown in Fig 8 can be used to describe this strategy layer. The initial link cost is set to be the distance between the adjacent cells and is dynamically updated using a penalized value that depends on the occupation of vehicle. It should be noted that the detour movement is determined by the combination effect of the desired route and the repulsive force from the conflicting vehicle.

As shown in Fig 8, the walking space is divided into separate cells where each cell is connected to each other by directed links. The size of each cell is usually set to 40×40 cm^2^ [11]. The initial link cost is set to the distance between the adjacent cells and is dynamically updated using a penalized value that depends on whether the cell is occupied by the turning vehicles. And then, a shortest path algorithm is applied to find the optimal route. It is assumed that pedestrians move with individual desired speeds along the optimal route. The desired moving direction is determined by the current position and the next node (center of the next cell) along the optimal route. Then, the driving force is updated as follows.

$${\vec{F}}_{d}=\frac{1}{\tau_{\alpha }}(v_{\alpha }^{d}{\vec{e}}_{detour}-{\vec{v}}_{\alpha })$$ (18)

where ${\vec{e}}_{detour}$ is the moving direction along the detour route.

**Fig 9:**
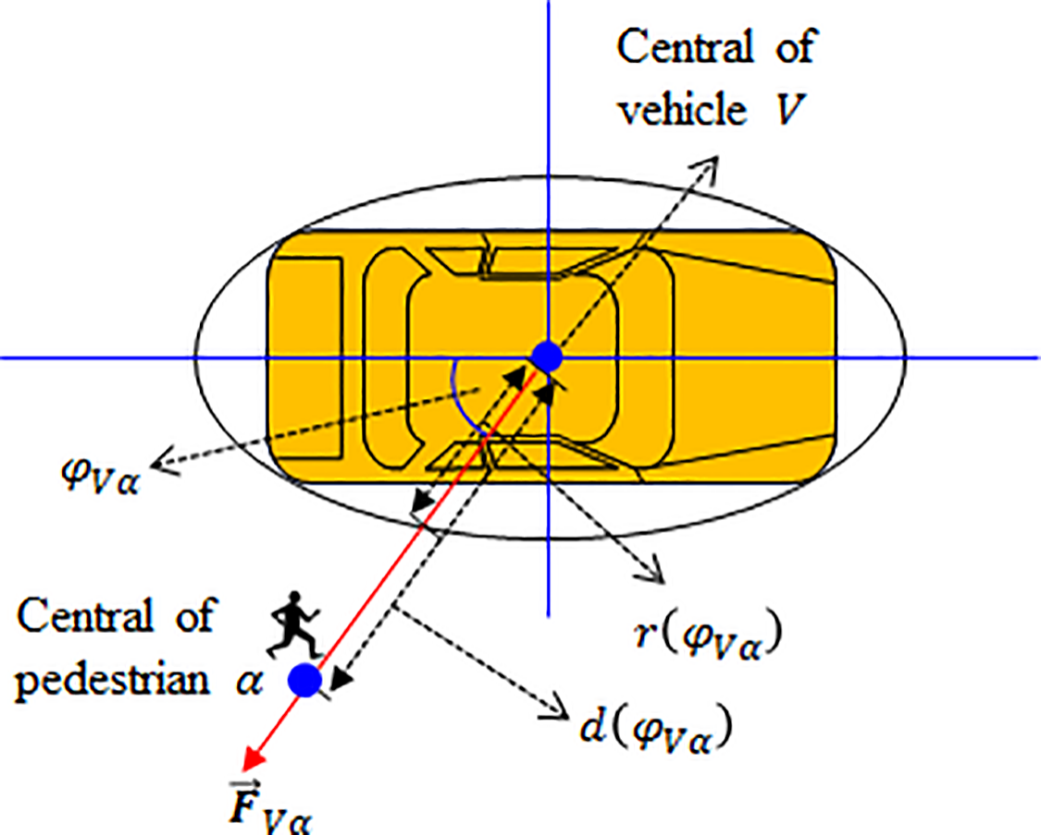
Lower layer: Repulsive force. A vehicle is now represented by an ellipse with the radius which depends on the angle between the moving direction of the vehicle and the moving direction of a close-by pedestrian.

In the lower strategy layer, the microscopic moving behavior is assumed to be affected by the vehicle force field once the vehicle enters the personal interaction range. Similar to the force from an obstacle, the repulsive force is determined by the pedestrian position and the direction of speed. Since the size of a vehicle is much larger than the pedestrian, it cannot be regarded as a point. As shown in Fig 9, a vehicle is now represented by an ellipse with the radius *r*(*φ*_*Vα*_) which depends on the angle between the moving direction of the vehicle and the moving direction of a close-by pedestrian. The radius can be formulated as follows.

$$r(\varphi_{V\alpha })=\frac{w}{1-(1-w^{2}/l^{2})\cos^{2}\left(\varphi_{V\alpha }\right)}$$ (19)

where *w* is the width of the turning vehicle and *l* is the length of the turning vehicle.

Accordingly, the repulsive force from a conflicting vehicle can be presented as follows.

$${\vec{F}}_{V\alpha }=\left\{ \begin{array}{l} A_{v}e^{\frac{r(\varphi_{V\alpha })-d(\varphi_{V\alpha })}{B_{v}}}{\vec{n}}_{V\alpha },\;if\;{\vec{v}}_{a}\cdot {\vec{n}}_{\alpha V}>0\\ 0,\;otherwise \end{array}\right.$$ (20)

where *d*(*φ*_*Vα*_) is the distance between the central of pedestrian *α* and the central of vehicle *V*, ${\vec{n}}_{V\alpha }$ is the normalized vector pointing from the central of pedestrian *V* and the central of vehicle *α*, and ${\vec{n}}_{\alpha V}$ is the normalized vector pointing from the central of pedestrian *α* and the central of vehicle *V*.

### Resultant force

The sum of the force terms exerted to pedestrian *α* from the desired destination, the counter-flow pedestrians, leading pedestrians, and turning vehicles can be expressed as follows.

$$\vec{F}(t_{k})={\vec{F}}_{d}(t_{k})+{\vec{F}}_{\alpha \beta }^{r}(t_{k})+{\vec{F}}_{tr}(t_{k})+{\vec{F}}_{V}(t_{k})+{\vec{F}}_{\varepsilon }$$ (21)

where $\vec{F}(t_{k})$ is the resultant force at time *t*_*k*_, ${\vec{F}}_{d}(t_{k})$ is the driving force at time *t*_*k*_, ${\vec{F}}_{\alpha \beta }^{r}(t_{k})$ is the repulsive force from conflicting pedestrian at time *t*_*k*_, ${\vec{F}}_{tr}(t_{k})$ is the attractive force from leading pedestrians at time *t*_*k*_, ${\vec{F}}_{V}(t_{k})$ is the repulsive force from conflicting vehicle at time *t*_*k*_, ${\vec{F}}_{\varepsilon }$ is the fluctuation term.

The step-wise speed and position can be expressed as follows:

$${\vec{v}}_{\alpha }(t_{k})={\vec{v}}_{\alpha }(t_{k-1})+\vec{F}(t_{k})\Delta t$$ (22)

$${\vec{P}}_{\alpha }(t_{k})={\vec{P}}_{\alpha }(t_{k-1})+{\vec{v}}_{\alpha }(t_{k})\Delta t+\frac{1}{2}{\vec{F}(t_{k})(\Delta t)}^{2}$$ (23)

where

${\vec{v}}_{\alpha }(t_{k})$: the updated speed at time *t*_*k*_;
${\vec{v}}_{\alpha }(t_{k-1})$: the previous speed at time *t*_*k*−1_;
Δ*t*: the simulation time step which is set to 0.04s in this study;
${\vec{P}}_{\alpha }(t_{k})$: the updated position at time *t*_*k*_;
${\vec{P}}_{\alpha }(t_{k-1})$: the previous position at time *t*_*k*−1_.

## Calibration methodology

**Fig 10:**
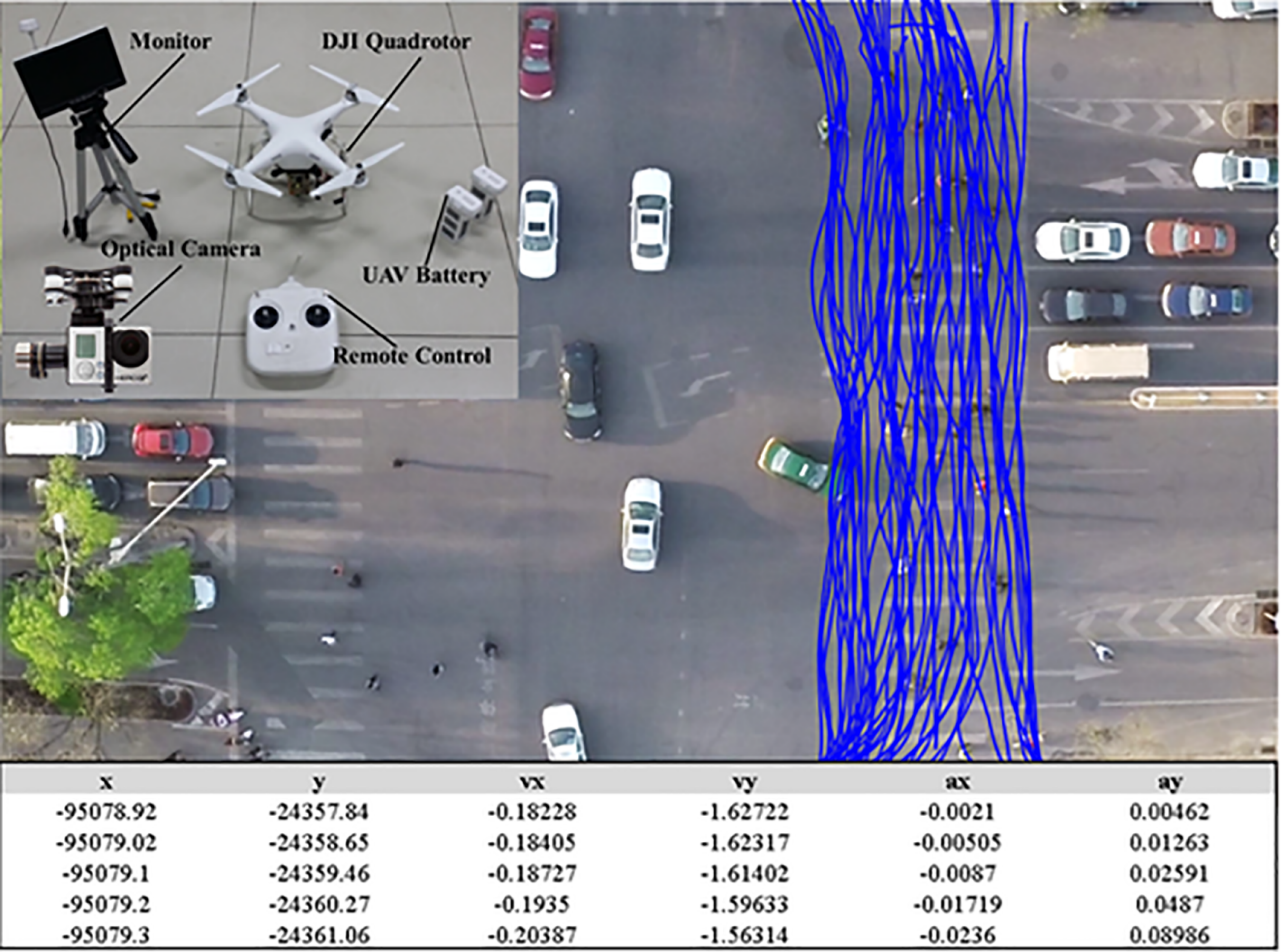
Collection of trajectories for model calibration. Empirical data were extracted using aerial videos captured by an optical camera with a 1920 × 1080 resolution mounted on a quadrotor with the flight altitude of about 40m-60m above the ground.

As shown in Fig 10, the empirical data were extracted using aerial videos captured by an optical camera with a 1920 × 1080 resolution mounted on a quadrotor with the flight altitude of about 40m-60m above the ground. The trajectories of pedestrians and turning vehicles at one intersection in Beijing, China were extracted from the video every 0.04s for model calibration. The dataset consists of the trajectories of 904 pedestrians and 156 turning vehicles. In total, 55,300 position samples are available. The available observations are trajectory profiles based on time series. From these data, all relevant quantities can be derived either directly or by applying finite differences, such as positions, velocities, accelerations, distances between pedestrians, and direction change.

To reproduce reasonable pedestrian trajectories in simulation, we calibrate the regression models (Eqs (4,5,8 and 15)) and the social force model (Eq (21)) based on maximum likelihood estimation (MLE). Because the social force model is two-dimensional (include x and y direction), a two-dimensional MLE [50] is introduced to calibration. The random error of Eq (21) is assumed to have a bivariate normal probabilistic density function with zero mean and a variance-covariance matrix (**Σ**). Accordingly, the likelihood *L*_*k*_ of a single prediction step is directly related to the probability density function of the normal distribution as follows.

$$L_{k}(\theta_{p})=\frac{1}{2\pi {\left|\Sigma \right|}^{1/2}}e^{-\frac{{\left(\vec{F}(t_{k},\theta_{p})-\vec{a}(t_{k})\right)}^{T}\Sigma^{-1}(\vec{F}(t_{k},\theta_{p})-\vec{a}(t_{k}))}{2}}$$ (24)

$$\theta_{p}=[\tau_{\alpha },A_{\beta }^{r},B_{\alpha \beta }^{r},A_{\beta }^{a},B_{\beta }^{a},A_{V},B_{V}]$$ (25)

where $\vec{a}(t_{k})$ is the observed acceleration at time *t*_*k*_, ***θ***_*p*_ the model parameters to be estimated.

For a set of *N* independent individuals and each individual has *M*_*i*_ time steps, the two-dimensional normal likelihood function can be formulated as follows.

$$L\left(\theta_{p}\right)=\prod_{i=1}^{N}\prod_{k=1}^{M_{i}}\frac{1}{2\pi {\left|\Sigma \right|}^{\frac{1}{2}}}e^{-\frac{{\left({\vec{F}}_{i}\left(t_{k},\theta_{p}\right)-{\vec{a}}_{i}\left(t_{k}\right)\right)}^{\text{T}}\Sigma^{-1}\left({\vec{F}}_{i}\left(t_{k},\theta_{p}\right)-{\vec{a}}_{i}\left(t_{k}\right)\right)}{2}}=\frac{1}{{\left(2\pi \right)}^{M_{1}M_{2}\cdots M_{N}}}\frac{1}{{\left|\Sigma \right|}^{M_{1}M_{2}\cdots M_{N}/2}}e^{-\frac{\sum_{i=1}^{N}\sum_{k=1}^{M_{i}}{\left({\vec{F}}_{i}\left(t_{k},\theta_{p}\right)-{\vec{a}}_{i}\left(t_{k}\right)\right)}^{\text{T}}\Sigma^{-1}\left({\vec{F}}_{i}\left(t_{k},\theta_{p}\right)-{\vec{a}}_{i}\left(t_{k}\right)\right)}{2}}$$ (26)

To facilitate the computation, the likelihood function is usually converted into a log-likelihood function as follows.

$$lnL(\theta_{p})=-M_{1}M_{2}\cdots M_{N}ln(2\pi )-\frac{M_{1}M_{2}\cdots M_{N}}{2}ln(\left|\Sigma \right|)-\frac{1}{2}\sum_{i=1}^{N}\sum_{k=1}^{M_{i}}{\left({\vec{F}}_{i}(t_{k},\theta_{p})-{\vec{a}}_{i}(t_{k})\right)}^{T}\Sigma^{-1}({\vec{F}}_{i}(t_{k},\theta_{p})-{\vec{a}}_{i}(t_{k}))$$ (27)

The maximum log-likelihood estimates of model parameters are obtained such that Eq (27) is maximized. This could be achieved by minimizing the negative log-likelihood, i.e., -*lnL*(***θ***_*p*_), in Matlab program. We use the “fminunc” function in Matlab to find a minimum of the negative log-likelihood function with several variables.

## Calibration result

Table 1 shows the parameter estimation for the distribution of exit position in Eqs (4) and (5). A parameter has statistical significance at a 95% confidence level if the p-value is less than 0.05. All of the parameters are statistically significant, indicating that the explanatory variables are meaningful. A positive sign of parameters means that the dependent variable increases as the explanatory variable value increases, while a negative sign means that the dependent variable decreases as the explanatory variable value increases. Interestingly, it is found that the increase in crosswalk length, crosswalk width and pedestrian density will lead to the increase of variation of exit position.

**Table 1:** Parameter estimation for exit position.

Equation	Variables	Description	Parameters	Estimates	p-value
Eq (4)	*M*_0_	A dummy denotes whether the OD direction is from N to F	*b*_0_	0.68	0.00
	*M*_1_	A dummy denotes whether the OD direction is straight	*b*_1_	-1.14	0.00
	*M*_2_	A dummy denotes whether the destination is on the stop line side	*b*_2_	-2.42	0.00
	*d*_*l*_	Crosswalk length	*b*_3_	-0.032	0.05
	*d*_*w*_	Crosswalk width	*b*_4_	0.11	0.03
	*ρ*	Pedestrian density	*b*_5_	1.98	0.01
		Constant	*b*_6_	5.33	0.00
Eq (5)	*M*_0_	A dummy denotes whether the OD direction is from N to F	*c*_0_	0.22	0.00
	*M*_1_	A dummy denotes whether the OD direction is straight	*c*_1_	-0.058	0.05
	*M*_2_	A dummy denotes whether the destination is on the stop line side	*c*_2_	-0.083	0.04
	*d*_*l*_	Crosswalk length	*c*_3_	0.00024	0.05
	*d*_*w*_	Crosswalk width	*c*_4_	0.062	0.03
	*ρ*	Pedestrian density	*c*_5_	1.70	0.00
		Constant	*c*_6_	0.86	0.05

Table 2 shows the parameter estimation for the desired speed expressed by Eqs (8 and 9). All of the parameters are statistically significant. This result indicates that the desired speed increases with increasing waiting time because the parameter sign for waiting time is positive, whereas the desired speed decreases with increasing pedestrian density because the parameter sign for pedestrian density is negative.

**Table 2:** Parameter estimation for desired speed.

Variable	Coefficient	Estimate	p-value
Waiting time (*t*_*w*_)	*a*_0_	0.0027	0.00
Pedestrian density (*ρ*)	*a*_1_	-0.56	0.00
Constant	*a*_2_	1.35	0.00
Standard deviation of error term	$\sigma_{\alpha }^{d}$	0.54	0.00

Table 3 shows the parameter estimation for acceleration behavior in Eqs (15–17) when a conflicting vehicle is approaching. The positive sign of *e*_0_ indicates that pedestrians tend to cross if the arrival time to the potential conflict point is earlier than that of the conflicting vehicle. In such a situation, the conflict with the vehicle might be alleviated if the vehicle maintains speeds or decelerates. However, the conflict becomes severe if the vehicle chooses to accelerate.

**Table 3:** Parameter estimation for “waiting/crossing” strategy.

Variables	Coefficient	Estimates	p-value
Relative time to the potential conflicting point when the pedestrian conflict with a moving vehicle (*T*_*ttc*_)	*e*_0_	0.031	0.00
Constant	*e*_1_	-0.46	0.00

Table 4 shows the calibration results for the social force model. The estimates are plausible in terms of their magnitude. According to the p-value at the 95% confidence level, all the parameters in the modified social force model are significant.

**Table 4:** Calibration results for social force model.

Parameters	Equation	Estimates	p-value
*τ*_*α*_	(6)	0.46	0.00
$A_{\beta }^{r}$	(13)	0.19	0.00
$B_{\alpha \beta }^{r}$	(13)	1.35	0.00
$A_{\beta }^{a}$	(14)	0.22	0.01
$B_{\beta }^{a}$	(14)	0.13	0.02
*A*_*V*_	(20)	0.93	0.02
*B*_*V*_	(20)	1.54	0.02

## Model Performance

### Error analysis

**Fig 11:**
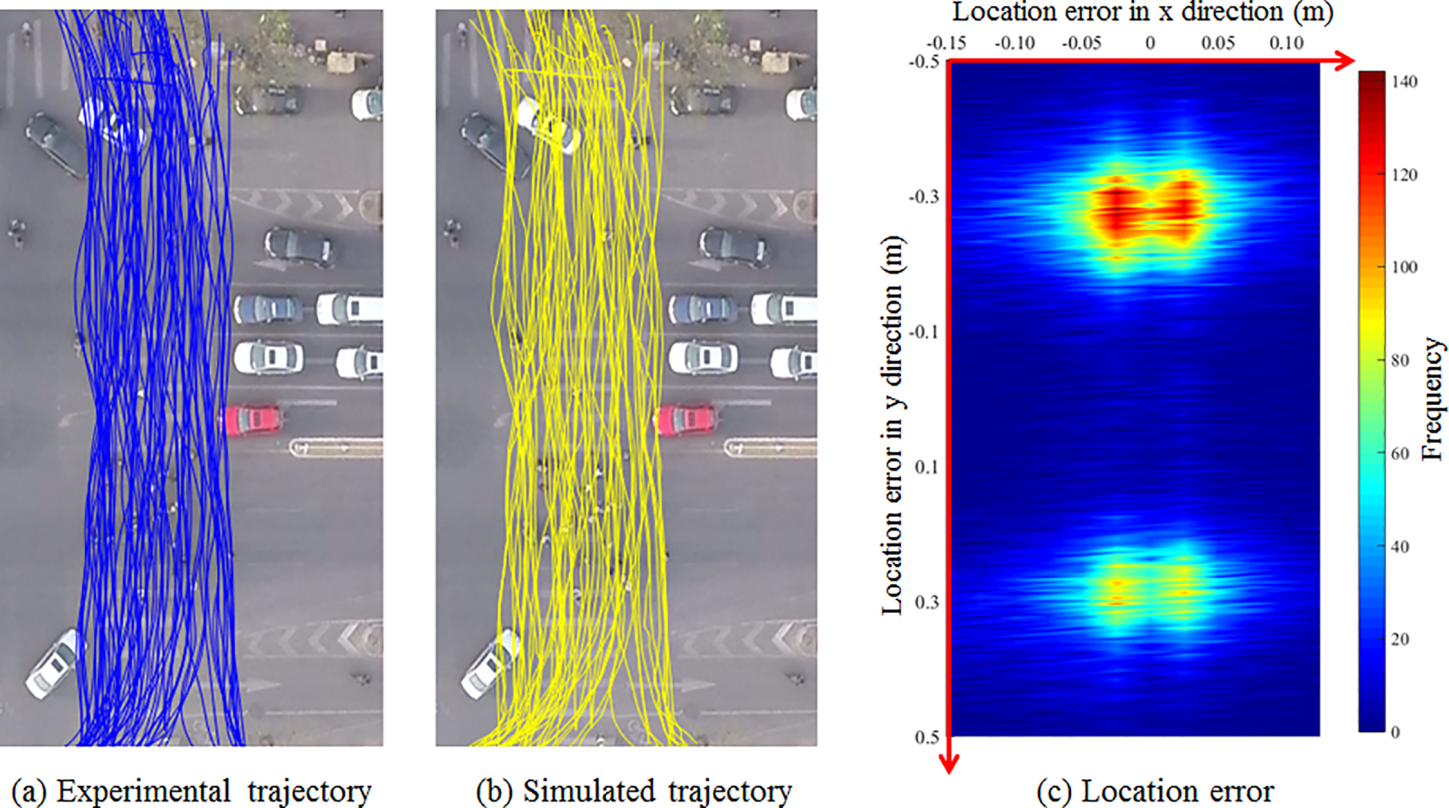
Trajectory comparison. (a) shows the pedestrian trajectories from real data and (b) shows simulation outputs.

**Fig 12:**
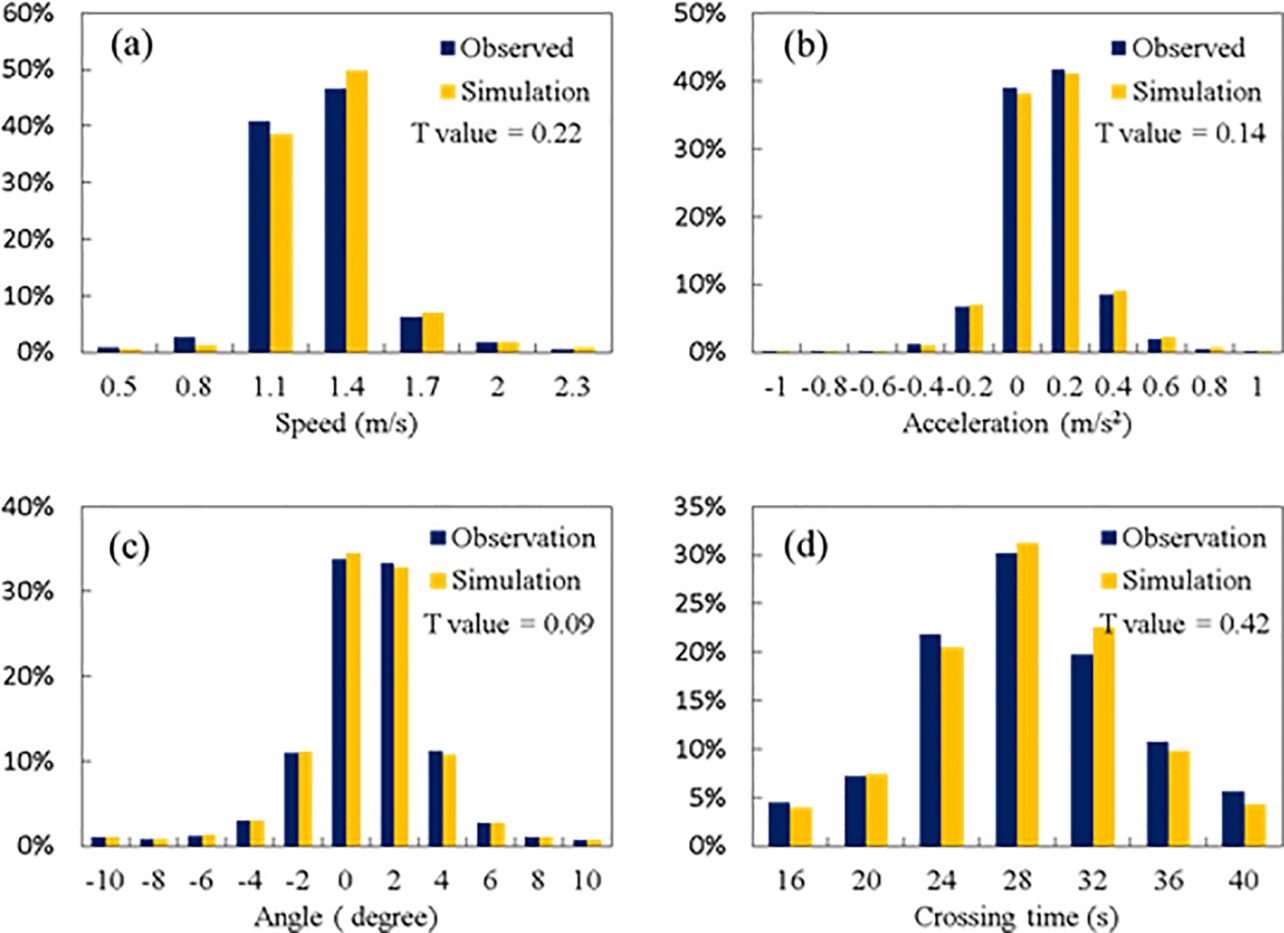
Simulation performance on speed, acceleration, direction change and crossing time. (a) shows the average absolute error of walking speed. (b) shows the comparison of step-wise acceleration distributions. (c) shows the distribution of the step-wise direction change between current and previous directions. (d) shows the distribution of the crossing time at crosswalk.

Fig 11(A) and 11(B) show the pedestrian trajectories from real data and simulation outputs, respectively. To quantify the trajectory performance, Fig 11(C) illustrates the location errors in x and y directions. It is found that 82.9% of the step-wise location errors are within 0.05m in x direction and 0.3m in y direction. To further illustrate the simulation performance, we compare the step-wise walking speed, step-wise acceleration, step-wise direction change and crossing time in observation and simulation. The comparison of the step-wise walking speed is shown in Fig 12(A). The average absolute error of walking speed in simulation is 0.16m/s. The speed distributions of the simulated environment match closely the observed data according to the t-value. Fig 12(B) shows the comparison of step-wise acceleration distributions. The observed and simulated values are not significantly different (t-value<1.96), which confirms the social force generated by the proposed model is reasonable. Fig 12(C) shows the distribution of the step-wise direction change between current and previous directions. Zero degree means the pedestrian keeps the current direction and move straight. The angle variation can reflect the frequency of interactions with conflicting pedestrians or other factors. According to the simulation results, most of the pedestrians keep their directions or only change a small angle in each time step, which agree with the statistics in the observed dataset. Fig 12 It was found that there are about 95% of the pedestrians finishing crossing within 36s. It is also consistent with the empirical observation.

### Fundamental diagram

**Fig 13:**
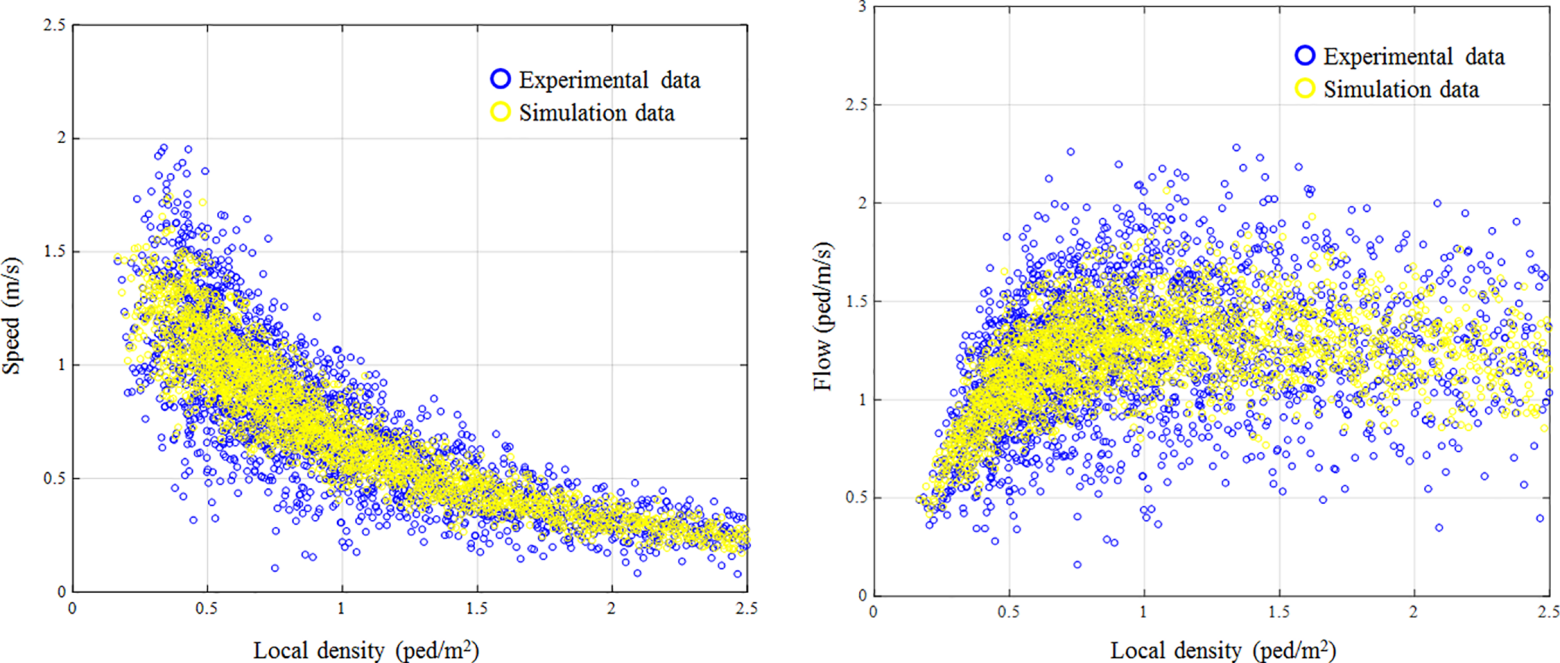
Fundamental diagrams of pedestrian flow. The simulated fundamental diagrams are in good agreement with the observed ones.

Since the pedestrian flow characteristic can be represented by fundamental diagram, we compare the speed-density and flow-density diagrams in real data and simulation outputs to demonstrate the model performance. The spatial mean speed and density are calculated by taking a cell 40×40 cm^2^ in the crosswalk as the measurement area. We calculate the speed and density every 1s. As shown in Fig 13, the simulated fundamental diagrams are in good agreement with the observed ones. But the scattered points are more scattered in observed data. It could be explained by the stochastic moving behavior because the moving direction and speed are quite flexible in the real situation especially in free-flow situation. However, the stochastic moving behavior has not been fully reproduced by simulation even though we applied a complex social force model and several behavior strategies. To improve the performance of fundamental diagram, the personal heterogeneity and stochasticity should be investigated in the future study.

### Lane formation

**Fig 14:**
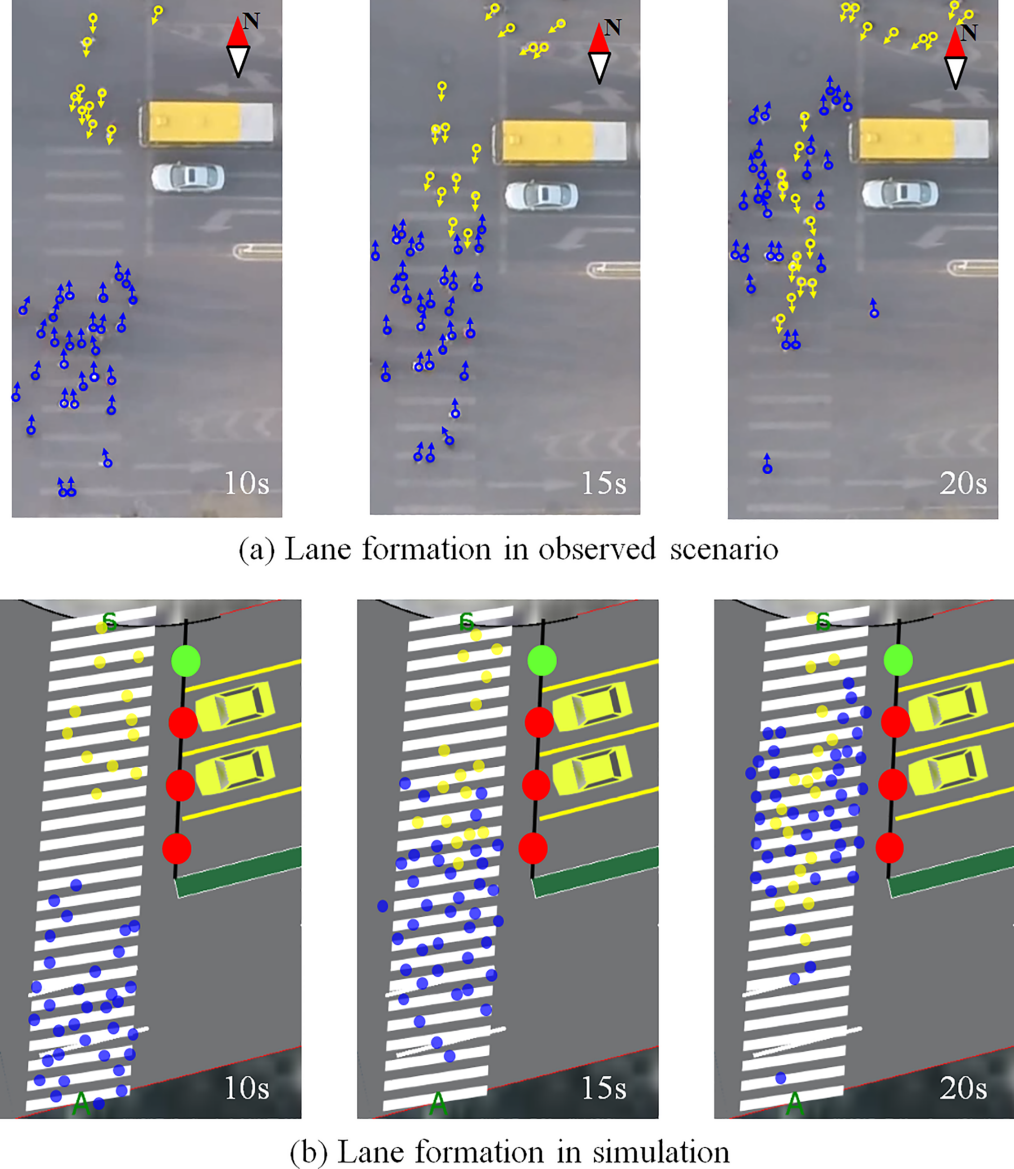
Lane formation phenomenon. The evolution of the lane formation in one signal cycle.

Lane formation is one of the most interesting phenomena that characterize pedestrian flow. Such a phenomenon is caused by conflict avoidance and leader-follower behavior. To demonstrate this phenomenon, a crowded scenario is set for the simulation. The bi-direction pedestrian demand is set to be 57 pedestrians per signal cycle according to the video recording. Fig 14 shows the evolution of the lane formation in one signal cycle. The snapshot at 10 s shows the bi-directional pedestrian flow without conflict with opposite pedestrians. At this moment, the lane formation phenomenon is not significant because the conflict from the front pedestrian is not intense. However, the southbound pedestrian flow begins to form a group when the pedestrians perceive a serious conflict from the counter-flow. The group formation can be explained by the attractive effect of the front “footprints”, which improves the smoothness when a large counter-flow crowd arrives. The snapshot at 15s shows the beginning of conflict occurrence. At this point, the lane formation phenomenon begins to occur. The lane formation from the north side appears first and can be explained by the observation that the pedestrian crowd with a smaller group size perceives the conflict earlier than the opposite pedestrian crowd with a larger group size. Earlier lane formation enables easier progress through the pedestrian counter-flow. The simulation shows that lane formation occurs when the bi-direction pedestrian flow meets, which is consistent with the observed scenario. The snapshot at 20 s shows that the “lane” is formed when the bi-directional pedestrian flow merges. The observed scenario shows that the southbound pedestrian flow forms one “lane”, and this “lane” separates the opposite flow into two “lanes”. In this manner, intensive interaction with the opposite pedestrians can be reduced, and a higher and more stable speed is possible. The “lanes” generated by the proposed model are consistent with the observed “lanes”. It was found that the “lanes” have a similar size and maintain a relatively stable shape for the crossing period using the proposed model.

## Conclusions and future work

A two-layer microscopic model is presented to simulate the interactions between pedestrians and vehicles at signalized intersections. A modified social force model considering the evasion behavior with counter-flow pedestrians, the following behavior with the leader pedestrians, and the collision avoidance behavior with vehicles was developed. The calibration is undertaken using the trajectory data (samples are given in S1 Table) of pedestrians and vehicles at one intersection in Beijing, China. The parameters of the developed model are calibrated by a two-dimensional MLE. Finally, the model performance is verified by comparing observed and estimated pedestrian flow characteristics, such as speed, acceleration, direction change, fundamental diagram and lane formation.

This simulation tool is recommended to be used by public authorities to gain more knowledge about how pedestrians and drivers interact with each other in the crosswalk. Furthermore, the cause of pedestrian-vehicle conflict can be identified in advance by simulation, which enables to provide information about potential safety problems prior to facility implementation.

## Supporting information

S1 Table Observed and simulated trajectory samples. (DOCX)
