# Supplementary material for: A microscopic simulation model for pedestrian-pedestrian and pedestrian-vehicle interactions at crosswalks
Source: PLoS One. 2017 Jul 17;12(7):e0180992. doi: 10.1371/journal.pone.0180992 (PMC5513439; doi:10.1371/journal.pone.0180992)
Supplement: S1 Table — (DOCX) [file pone.0180992.s001.docx]

S1 Table Observed and simulated trajectory samples

| id | x_obs | y_obs | x_est | y_est |
| --- | --- | --- | --- | --- |
| 1 | 2.57 | 38.10 | 2.57 | 38.10 |
| 1 | 2.78 | 37.39 | 2.78 | 37.39 |
| 1 | 2.95 | 36.69 | 2.95 | 36.69 |
| 1 | 3.11 | 35.97 | 3.11 | 35.97 |
| 1 | 3.27 | 35.22 | 3.27 | 35.22 |
| 1 | 3.41 | 34.42 | 3.25 | 33.72 |
| 1 | 3.52 | 33.57 | 3.15 | 33.34 |
| 1 | 3.60 | 32.69 | 3.10 | 32.11 |
| 1 | 3.63 | 31.79 | 3.09 | 31.78 |
| 1 | 3.62 | 30.90 | 3.02 | 30.86 |
| 1 | 3.56 | 30.05 | 2.98 | 29.71 |
| 1 | 3.50 | 29.22 | 2.94 | 28.90 |
| 1 | 3.44 | 28.42 | 2.85 | 28.00 |
| 1 | 3.38 | 27.61 | 2.83 | 27.23 |
| 1 | 3.31 | 26.80 | 2.75 | 26.57 |
| 1 | 3.22 | 25.98 | 2.73 | 25.39 |
| 1 | 3.12 | 25.17 | 2.66 | 24.91 |
| 1 | 2.99 | 24.36 | 2.62 | 23.85 |
| 1 | 2.84 | 23.56 | 2.58 | 23.59 |
| 1 | 2.66 | 22.76 | 2.54 | 22.65 |
| 1 | 2.51 | 21.96 | 2.48 | 21.50 |
| 1 | 2.37 | 21.14 | 2.41 | 20.47 |
| 1 | 2.27 | 20.30 | 2.38 | 19.66 |
| 1 | 2.23 | 19.45 | 2.32 | 18.97 |
| 1 | 2.23 | 18.61 | 2.28 | 18.68 |
| 1 | 2.28 | 17.78 | 2.23 | 17.60 |
| 1 | 2.34 | 16.95 | 2.16 | 16.82 |
| 1 | 2.42 | 16.13 | 2.13 | 16.15 |
| 1 | 2.48 | 15.32 | 2.07 | 15.39 |
| 1 | 2.52 | 14.52 | 2.00 | 13.79 |
| 1 | 2.50 | 13.72 | 1.97 | 13.20 |
| 1 | 2.45 | 12.92 | 1.92 | 12.82 |
| 1 | 2.37 | 12.11 | 1.85 | 11.71 |
| 1 | 2.27 | 11.28 | 1.80 | 10.77 |
| 1 | 2.16 | 10.42 | 1.79 | 10.17 |
| 1 | 2.07 | 9.53 | 1.71 | 9.16 |
| 1 | 1.99 | 8.64 | 1.65 | 8.48 |
| 1 | 1.94 | 7.77 | 1.64 | 7.51 |
| 1 | 1.91 | 6.97 | 1.56 | 6.28 |
| 1 | 1.88 | 6.23 | 1.51 | 5.84 |
| 1 | 1.86 | 5.52 | 1.49 | 5.28 |
| 1 | 1.81 | 4.83 | 1.42 | 4.45 |
| 1 | 1.74 | 4.11 | 1.36 | 3.31 |
| 1 | 1.65 | 3.36 | 1.32 | 2.11 |
| 1 | 1.52 | 2.57 | 1.29 | 1.88 |
| 1 | 1.38 | 1.74 | 1.24 | 0.83 |
| 1 | 1.21 | 0.86 | 1.17 | -0.09 |
| 1 | 1.04 | -0.08 | 1.09 | -0.17 |
| 1 | 0.87 | -1.11 | 1.01 | -1.24 |
| 1 | 0.71 | -2.23 | 0.93 | -2.50 |
| 1 | 0.56 | -3.45 | 0.57 | -3.03 |
| 2 | -0.20 | 37.63 | -0.17 | 37.63 |
| 2 | 0.15 | 37.24 | 0.08 | 36.67 |
| 2 | 0.38 | 36.73 | 0.32 | 36.04 |
| 2 | 0.60 | 36.10 | 0.56 | 35.44 |
| 2 | 0.77 | 35.40 | 0.79 | 34.98 |
| 2 | 0.89 | 34.65 | 0.85 | 34.65 |
| 2 | 0.95 | 33.89 | 0.91 | 34.35 |
| 2 | 0.98 | 33.11 | 0.87 | 33.70 |
| 2 | 0.95 | 32.34 | 0.92 | 33.05 |
| 2 | 0.88 | 31.58 | 0.85 | 32.40 |
| 2 | 0.81 | 30.86 | 0.85 | 31.75 |
| 2 | 0.74 | 30.18 | 0.93 | 31.11 |
| 2 | 0.69 | 29.54 | 0.93 | 30.46 |
| 2 | 0.66 | 28.94 | 0.91 | 29.81 |
| 2 | 0.65 | 28.35 | 0.87 | 29.16 |
| 2 | 0.64 | 27.77 | 0.89 | 28.51 |
| 2 | 0.63 | 27.18 | 0.91 | 27.86 |
| 2 | 0.61 | 26.57 | 0.94 | 27.21 |
| 2 | 0.58 | 25.93 | 0.85 | 26.56 |
| 2 | 0.55 | 25.27 | 0.94 | 25.91 |
| 2 | 0.53 | 24.63 | 0.86 | 25.26 |
| 2 | 0.54 | 24.00 | 0.85 | 24.61 |
| 2 | 0.58 | 23.39 | 0.93 | 23.96 |
| 2 | 0.60 | 22.80 | 0.94 | 23.32 |
| 2 | 0.65 | 22.21 | 0.94 | 22.67 |
| 2 | 0.68 | 21.63 | 0.88 | 22.02 |
| 2 | 0.70 | 21.06 | 0.87 | 21.37 |
| 2 | 0.72 | 20.50 | 0.85 | 20.72 |
| 2 | 0.75 | 19.93 | 0.87 | 20.07 |
| 2 | 0.78 | 19.36 | 0.88 | 19.42 |
| 2 | 0.82 | 18.75 | 0.88 | 18.77 |
| 2 | 0.85 | 18.12 | 0.91 | 18.12 |
| 2 | 0.91 | 17.46 | 0.93 | 17.47 |
| 2 | 0.92 | 16.79 | 0.90 | 16.82 |
| 2 | 0.93 | 16.11 | 0.88 | 16.17 |
| 2 | 0.94 | 15.44 | 0.89 | 15.53 |
| 2 | 0.96 | 14.79 | 0.94 | 14.88 |
| 2 | 1.02 | 14.13 | 0.91 | 14.23 |
| 2 | 1.05 | 13.47 | 0.96 | 13.58 |
| 2 | 1.07 | 12.80 | 0.98 | 12.93 |
| 2 | 1.13 | 12.15 | 0.89 | 12.28 |
| 2 | 1.18 | 11.50 | 0.92 | 11.63 |
| 2 | 1.23 | 10.87 | 1.02 | 10.98 |
| 2 | 1.28 | 10.25 | 0.97 | 10.50 |
| 2 | 1.32 | 9.63 | 1.15 | 10.10 |
| 2 | 1.35 | 9.00 | 1.28 | 9.40 |
| 2 | 1.37 | 8.37 | 1.32 | 8.90 |
| 2 | 1.38 | 7.71 | 1.38 | 8.10 |
| 2 | 1.38 | 7.03 | 1.47 | 7.50 |
| 2 | 1.35 | 6.34 | 1.51 | 6.80 |
| 2 | 1.31 | 5.62 | 1.48 | 6.10 |
| 2 | 1.27 | 4.89 | 1.48 | 5.40 |
| 2 | 1.20 | 4.16 | 1.51 | 4.60 |
| 2 | 1.18 | 3.44 | 1.44 | 3.84 |
| 2 | 1.15 | 2.73 | 1.39 | 3.19 |
| 2 | 1.12 | 2.04 | 1.30 | 2.54 |
| 2 | 1.08 | 1.36 | 1.20 | 1.89 |
| 2 | 1.02 | 0.70 | 1.00 | 0.70 |
| 2 | 0.99 | 0.04 | 0.91 | 0.02 |
| 2 | 0.99 | 0.04 | 0.91 | 0.02 |
| 2 | 0.99 | 0.04 | 0.91 | 0.02 |
| 3 | 1.88 | 35.22 | 2.10 | 35.18 |
| 3 | 1.95 | 34.75 | 1.95 | 34.75 |
| 3 | 2.03 | 34.16 | 2.10 | 34.22 |
| 3 | 2.12 | 33.46 | 2.05 | 33.42 |
| 3 | 2.20 | 32.66 | 1.97 | 32.58 |
| 3 | 2.29 | 31.83 | 1.92 | 31.80 |
| 3 | 2.38 | 31.01 | 1.95 | 30.82 |
| 3 | 2.45 | 30.22 | 2.12 | 30.08 |
| 3 | 2.53 | 29.48 | 2.23 | 29.36 |
| 3 | 2.62 | 28.77 | 2.31 | 28.67 |
| 3 | 2.71 | 28.10 | 2.50 | 27.88 |
| 3 | 2.81 | 27.46 | 2.55 | 27.38 |
| 3 | 2.89 | 26.87 | 2.51 | 26.67 |
| 3 | 2.98 | 26.32 | 2.62 | 26.23 |
| 3 | 3.06 | 25.78 | 2.71 | 25.76 |
| 3 | 3.13 | 25.25 | 2.80 | 25.17 |
| 3 | 3.19 | 24.69 | 2.91 | 24.55 |
| 3 | 3.23 | 24.10 | 2.96 | 23.91 |
| 3 | 3.24 | 23.44 | 3.12 | 23.25 |
| 3 | 3.23 | 22.70 | 3.32 | 22.70 |
| 3 | 3.19 | 21.91 | 3.42 | 21.87 |
| 3 | 3.12 | 21.07 | 3.45 | 21.04 |
| 3 | 3.05 | 20.20 | 3.21 | 20.22 |
| 3 | 2.98 | 19.34 | 3.15 | 19.29 |
| 3 | 2.97 | 18.47 | 2.95 | 18.45 |
| 3 | 3.01 | 17.57 | 2.85 | 17.50 |
| 3 | 3.09 | 16.60 | 2.92 | 16.69 |
| 3 | 3.18 | 15.57 | 3.01 | 15.57 |
| 3 | 3.27 | 14.51 | 3.10 | 14.58 |
| 3 | 3.31 | 13.47 | 3.20 | 13.42 |
| 3 | 3.27 | 12.54 | 3.19 | 12.38 |
| 3 | 3.12 | 11.74 | 3.11 | 11.50 |
| 3 | 2.88 | 11.04 | 2.88 | 10.86 |
| 3 | 2.56 | 10.42 | 2.68 | 10.22 |
| 3 | 2.20 | 9.79 | 2.45 | 9.77 |
| 3 | 1.83 | 9.12 | 2.15 | 8.93 |
| 3 | 1.49 | 8.37 | 2.19 | 8.40 |
| 3 | 1.22 | 7.57 | 1.85 | 7.57 |
| 3 | 1.02 | 6.77 | 1.54 | 6.63 |
| 3 | 0.91 | 6.00 | 1.22 | 6.00 |
| 3 | 0.88 | 5.30 | 0.95 | 5.03 |
| 3 | 0.91 | 4.64 | 0.88 | 4.57 |
| 3 | 0.98 | 3.98 | 0.81 | 3.97 |
| 3 | 1.02 | 3.27 | 0.75 | 3.16 |
| 3 | 1.03 | 2.50 | 0.71 | 2.41 |
| 3 | 0.98 | 1.66 | 0.82 | 1.64 |
| 3 | 0.90 | 0.78 | 0.78 | 0.85 |
| 3 | 0.79 | -0.11 | 0.75 | -0.16 |
| 3 | 0.79 | -0.11 | 0.75 | -0.16 |
| 4 | 0.24 | 37.66 | 0.24 | 37.85 |
| 4 | 0.44 | 35.76 | 0.36 | 36.00 |
| 4 | 0.55 | 33.93 | 0.40 | 34.05 |
| 4 | 0.59 | 32.18 | 0.50 | 32.46 |
| 4 | 0.61 | 30.51 | 0.68 | 30.66 |
| 4 | 0.65 | 28.90 | 0.70 | 29.11 |
| 4 | 0.72 | 27.35 | 0.80 | 27.31 |
| 4 | 0.81 | 25.86 | 0.75 | 26.05 |
| 4 | 0.90 | 24.43 | 0.86 | 24.26 |
| 4 | 1.00 | 23.06 | 0.85 | 23.16 |
| 4 | 1.13 | 21.73 | 0.95 | 21.82 |
| 4 | 1.30 | 20.41 | 1.12 | 20.61 |
| 4 | 1.55 | 19.08 | 1.40 | 18.92 |
| 4 | 1.89 | 17.74 | 1.55 | 17.97 |
| 4 | 2.32 | 16.35 | 1.80 | 16.37 |
| 4 | 2.78 | 14.92 | 2.20 | 15.16 |
| 4 | 3.22 | 13.46 | 2.80 | 13.56 |
| 4 | 3.56 | 11.99 | 3.20 | 11.95 |
| 4 | 3.77 | 10.54 | 3.30 | 10.72 |
| 4 | 3.82 | 9.16 | 3.50 | 9.18 |
| 4 | 3.75 | 7.88 | 3.40 | 7.71 |
| 4 | 3.59 | 6.74 | 3.20 | 6.45 |
| 4 | 3.40 | 5.73 | 3.10 | 5.73 |
| 4 | 3.20 | 4.85 | 2.90 | 4.52 |
| 4 | 2.98 | 4.06 | 2.70 | 3.84 |
| 4 | 2.79 | 3.32 | 2.60 | 3.31 |
| 4 | 2.60 | 2.20 | 2.55 | 2.49 |
| 4 | 2.40 | 1.60 | 2.30 | 0.90 |
| 4 | 2.00 | 0.90 | 1.90 | 0.40 |
| 4 | 1.50 | 0.50 | 1.60 | 0.00 |
| 4 | 1.45 | -0.30 | 1.45 | 0.00 |
| 5 | 1.38 | -0.09 | 1.38 | -0.09 |
| 5 | 1.38 | -0.09 | 1.38 | -0.09 |
| 5 | 1.41 | 0.57 | 1.25 | 0.60 |
| 5 | 1.45 | 1.15 | 1.23 | 1.13 |
| 5 | 1.47 | 1.67 | 1.20 | 1.60 |
| 5 | 1.51 | 2.19 | 1.30 | 2.01 |
| 5 | 1.56 | 2.71 | 1.45 | 2.88 |
| 5 | 1.61 | 3.26 | 1.35 | 3.34 |
| 5 | 1.65 | 3.84 | 1.40 | 3.92 |
| 5 | 1.68 | 4.45 | 1.35 | 4.51 |
| 5 | 1.70 | 5.09 | 1.42 | 5.06 |
| 5 | 1.70 | 5.75 | 1.52 | 5.77 |
| 5 | 1.70 | 6.40 | 1.52 | 6.27 |
| 5 | 1.70 | 7.05 | 1.55 | 7.10 |
| 5 | 1.72 | 7.68 | 1.45 | 7.63 |
| 5 | 1.76 | 8.30 | 1.50 | 8.09 |
| 5 | 1.81 | 8.93 | 1.60 | 8.82 |
| 5 | 1.87 | 9.58 | 1.80 | 9.54 |
| 5 | 1.92 | 10.23 | 1.90 | 10.09 |
| 5 | 1.95 | 10.88 | 2.10 | 10.71 |
| 5 | 1.97 | 11.51 | 2.22 | 11.58 |
| 5 | 1.96 | 12.11 | 2.30 | 12.24 |
| 5 | 1.93 | 12.68 | 2.40 | 12.78 |
| 5 | 1.90 | 13.23 | 2.50 | 13.18 |
| 5 | 1.87 | 13.78 | 2.45 | 13.93 |
| 5 | 1.84 | 14.32 | 2.32 | 14.49 |
| 5 | 1.82 | 14.87 | 2.21 | 14.94 |
| 5 | 1.77 | 15.42 | 2.10 | 15.75 |
| 5 | 1.71 | 15.98 | 2.05 | 15.98 |
| 5 | 1.63 | 16.54 | 1.92 | 16.75 |
| 5 | 1.54 | 17.11 | 1.95 | 17.25 |
| 5 | 1.46 | 17.70 | 1.90 | 17.63 |
| 5 | 1.40 | 18.30 | 1.85 | 18.33 |
| 5 | 1.38 | 18.90 | 1.80 | 19.27 |
| 5 | 1.39 | 19.50 | 1.71 | 19.55 |
| 5 | 1.44 | 20.11 | 1.72 | 20.15 |
| 5 | 1.50 | 20.70 | 1.73 | 20.79 |
| 5 | 1.57 | 21.30 | 1.73 | 21.25 |
| 5 | 1.64 | 21.90 | 1.78 | 21.96 |
| 5 | 1.70 | 22.49 | 1.95 | 22.68 |
| 5 | 1.74 | 23.08 | 1.92 | 23.27 |
| 5 | 1.77 | 23.67 | 2.03 | 23.73 |
| 5 | 1.80 | 24.28 | 2.10 | 24.24 |
| 5 | 1.81 | 24.89 | 2.05 | 24.76 |
| 5 | 1.82 | 25.50 | 2.20 | 25.53 |
| 5 | 1.83 | 26.11 | 2.10 | 26.05 |
| 5 | 1.84 | 26.71 | 2.15 | 26.74 |
| 5 | 1.86 | 27.31 | 2.20 | 27.31 |
| 5 | 1.88 | 27.92 | 2.34 | 27.74 |
| 5 | 1.88 | 28.54 | 2.30 | 28.42 |
| 5 | 1.88 | 29.18 | 2.20 | 29.10 |
| 5 | 1.87 | 29.82 | 2.00 | 29.78 |
| 5 | 1.84 | 30.47 | 1.95 | 30.66 |
| 5 | 1.81 | 31.11 | 1.90 | 31.00 |
| 5 | 1.74 | 31.71 | 1.85 | 31.76 |
| 5 | 1.66 | 32.27 | 1.79 | 32.33 |
| 5 | 1.60 | 33.00 | 1.74 | 33.23 |
| 5 | 1.52 | 33.50 | 1.72 | 33.41 |
| 5 | 1.50 | 34.30 | 1.70 | 34.45 |
| 5 | 1.60 | 34.90 | 1.61 | 34.90 |
| 5 | 1.42 | 35.71 | 1.42 | 36.00 |
